# Preferential Duplication of Intermodular Hub Genes: An Evolutionary Signature in Eukaryotes Genome Networks

**DOI:** 10.1371/journal.pone.0056579

**Published:** 2013-02-26

**Authors:** Ricardo M. Ferreira, José Luiz Rybarczyk-Filho, Rodrigo J. S. Dalmolin, Mauro A. A. Castro, José C. F. Moreira, Leonardo G. Brunnet, Rita M. C. de Almeida

**Affiliations:** 1 Instituto de Física, Universidade Federal do Rio Grande do Sul, Porto Alegre, Brazil; 2 National Institute of Science and Technology for Complex Systems, Universidade Federal do Rio Grande do Sul, Porto Alegre, Brazil; 3 Departamento de Bioquímica, Universidade Federal do Rio Grande do Sul, Porto Alegre, Brazil; University of Georgia, United States of America

## Abstract

Whole genome protein-protein association networks are not random and their topological properties stem from genome evolution mechanisms. In fact, more connected, but less clustered proteins are related to genes that, in general, present more paralogs as compared to other genes, indicating frequent previous gene duplication episodes. On the other hand, genes related to conserved biological functions present few or no paralogs and yield proteins that are highly connected and clustered. These general network characteristics must have an evolutionary explanation. Considering data from STRING database, we present here experimental evidence that, more than not being scale free, protein degree distributions of organisms present an increased probability for high degree nodes. Furthermore, based on this experimental evidence, we propose a simulation model for genome evolution, where genes in a network are either acquired *de novo* using a preferential attachment rule, or duplicated with a probability that linearly grows with gene degree and decreases with its clustering coefficient. For the first time a model yields results that simultaneously describe different topological distributions. Also, this model correctly predicts that, to produce protein-protein association networks with number of links and number of nodes in the observed range for Eukaryotes, it is necessary 90% of gene duplication and 10% of *de novo* gene acquisition. This scenario implies a universal mechanism for genome evolution.

## Introduction

Genome evolution is determined first by the processes that modify DNA and then by those mechanisms that either neutrally keep or naturally select these mutations by their phenotypic effects. The connection between DNA variations and the consequent phenotypic alterations is far from being simple and is elusive to determine. However, it is reasonable to assume that, after evolutionary time spans, these DNA variation mechanisms have left their mark on the genome.

Phenotypic effects are consequence of the existing associations between proteins which rule cellular metabolism. As proteins are expressed from genes, protein-protein associations will express eventual changes in genotypes and are prone to natural selection. Consequently we may speculate that natural selection, by defining genome evolution mechanisms, has left its mark on organisms’ protein-protein association matrices. This is not a novel idea. Barabási and collaborators [1], [2] have described genomes of different organisms as networks where nodes are either genes or proteins, and links correspond to associations between the nodes. They proposed an evolution dynamics for the genome considering that genes are sequentially added to a network following a preferential attachment rule: each newly incorporated gene interacts with a gene already on the network with a probability that is proportional to its degree, that is, to the number of other genes with which it already interacts. The resulting artificial network is scale free and described well the available experimental data at that date.

However, the properties of a gene already in the network are not the only driving force for a novel gene attachment. There are different molecular mechanisms acting as novelty source in gene formation, such as exon shuffling, retroposition, mobile elements, horizontal gene transfer, gene duplication, etc., and the connections of a new gene certainly reflect its origin together with the nature of the genes it connects to [3]. Although the relevance of these mechanisms differs between Eukaryotes and Prokaryotes, gene duplication is recognizably the most important and there is plenty of evidence that it plays an essential role on genome evolution [4]. One major feature of a duplicated gene consists of inheriting its parent connections and this property is determinant to the whole network design.

Vázquez and collaborators [5], [6] proposed a model for genome evolution where genes are incorporated by duplication followed by mutations which are translated as adding and/or deleting links on a protein-protein association matrix. In this model, genes are randomly chosen to duplicate and parameters are set to produce gene networks where the probability that a gene product is associated to 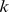 other proteins decays as a power law as 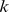 increases. A drawback for this approach, using randomly chosen genes, lays on the experimental fact that the probability to fix a given duplication episode greatly varies according to the properties of the duplicating gene [7]–[9].

Since the contributions by Barabási and collaborators, the amount and quality of data regarding both genomes and protein-protein association have greatly increased. For example, STRING database increased from few organisms at 2001 to 1133 organisms in 2011 [10]–[12]. Also, databases regarding protein-protein association for some organisms have been largely enhanced. Here we analyze data considering 31 core eukaryote organisms, which strongly suggest that highly connected genes stem from duplication mechanisms acting preferentially on genes that are highly connected, but not excessively clustered. These conclusions are made evident here by presenting the quantities as functions of 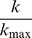, where 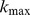 is the maximum degree in the network. We also propose an adequate ordering for genes to globally illustrate topological properties of the protein-protein association matrix.

Considering these conclusions based on the information provided by STRING database we propose a genome evolution dynamics where the probability that a gene duplicates grows with its degree and decreases depending on how clustered it is. We also consider a Barabási mechanism of acquiring genes *de novo* based on preferential attachment. The results of these simulations are capable of describing different aspects of the network topology, besides predicting the ratio of duplicated and *de novo* acquired genes.

## Results

### Building Protein-protein Association Matrices

Many different gene networks may be built, depending on how nodes and links are defined. Regulatory gene networks, for example, consider regulation between genes to assign gene association [13], metabolic pathways may be represented by graphs where the direct interaction between gene products stand for links [14], or gene co-expression may be taken into account when specifying the connections in a gene network. Depending on how the network is built, genes present different network properties as their degree, clustering coefficient, centrality, etc.

Here we investigate the marks that evolution by natural selection has left on the topology of a gene network. Natural selection acts on the organism phenotype that is strongly defined by the organism genotype. Advantageous gene product associations have certainly been selected, and probably the topology of these associations are a consequence of this selection pressure. Consequently, the gene network we must consider here is a network whose links may be acted on by natural selection. As virtually all kinds of associations between genes or gene products may end up with a phenotypic consequence, we must consider all kinds of associations. Furthermore, we will consider a genome evolution model describing only those genes that have already been selected to be conserved for evolutionary time spans.Building a gene network with all kinds of gene association (or gene product association) implies an extensive amount of work, which is only made possible by integrating the results obtained by different scientists, laboratories, and techniques. The techniques vary from very accurate ones, where the evidence for a gene-gene association has been observed *in vivo* by different laboratories, to high-throughput experiments where, for example, many gene products are assessed simultaneously in solutions that are not simulating the interior of a cell. Also there may be predicted gene-gene association, based on computational inference by similarity between gene products in different organisms. Such a large diversity of techniques to assign gene-gene association leads to a variable confidence degree in the trueness of the results, and a confidence scoring is a necessary tool. The problem resides in controlling the degree of false positives, which is too high when all high-throughput evidence is considered, and of false negatives, which is too high when only evidence from very accurate experiments are taken into account.

Fortunately, STRING provides an integrated database for gene-gene association that considers different organisms and kinds of evidence, with the required control of a confidence score. Other databases yield different gene networks, but they either *i)* present a small number of organisms as compared with STRING, *ii)* assume specific criteria for assigning gene-gene associations (as only gene regulation [13] or only physical evidence of protein-protein association [15]) and/or *iii)* do not provide a confidence scoring aiming at false positives control. On the other hand, after choosing STRING as the adequate database, it is still necessary to choose the adequate value for the confidence score.

For that we produced gene networks for all 31 core eukaryote organisms in STRING database, version 8.3 [10]–[12], with confidence scores 0.400, 0.500, 0.600, and 0.700, 0.800, and 0.900 using “experimental” and “database” (95% of these interactions) added with “neighborhood”, “fusion”, “co-expression”, and “co-occurrence” evidence. Text S1 we discuss STRING confidence score and present a plot of number of links versus number of nodes considering all confidence scores for the six most studied eukaryotes: there is a sudden drop on the curves as the confidence scores grows from 0.800 to 0.900, signaling that many links are discarded at that point. We chose to work with confidence scores from 0.700 to 0.900.

Gene networks are built such that each node corresponds to a protein with at least one known protein-protein association, and links correspond to these associations. To each network node 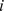 we assign a degree 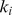, which is its the number of links. For each organism and score we produce a network and calculate the probability 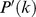 that a protein has 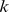 links, defined as
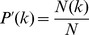
(1)where *N* is the number of nodes and 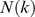 is the number of nodes with degree 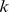. To compare different organisms, with different genome sizes, we considered a rescaled degree to obtain the probability of finding a protein with a given degree *k*, as follows
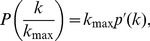
(2)where 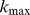 is the maximum degree in that network.

**Figure 1:**
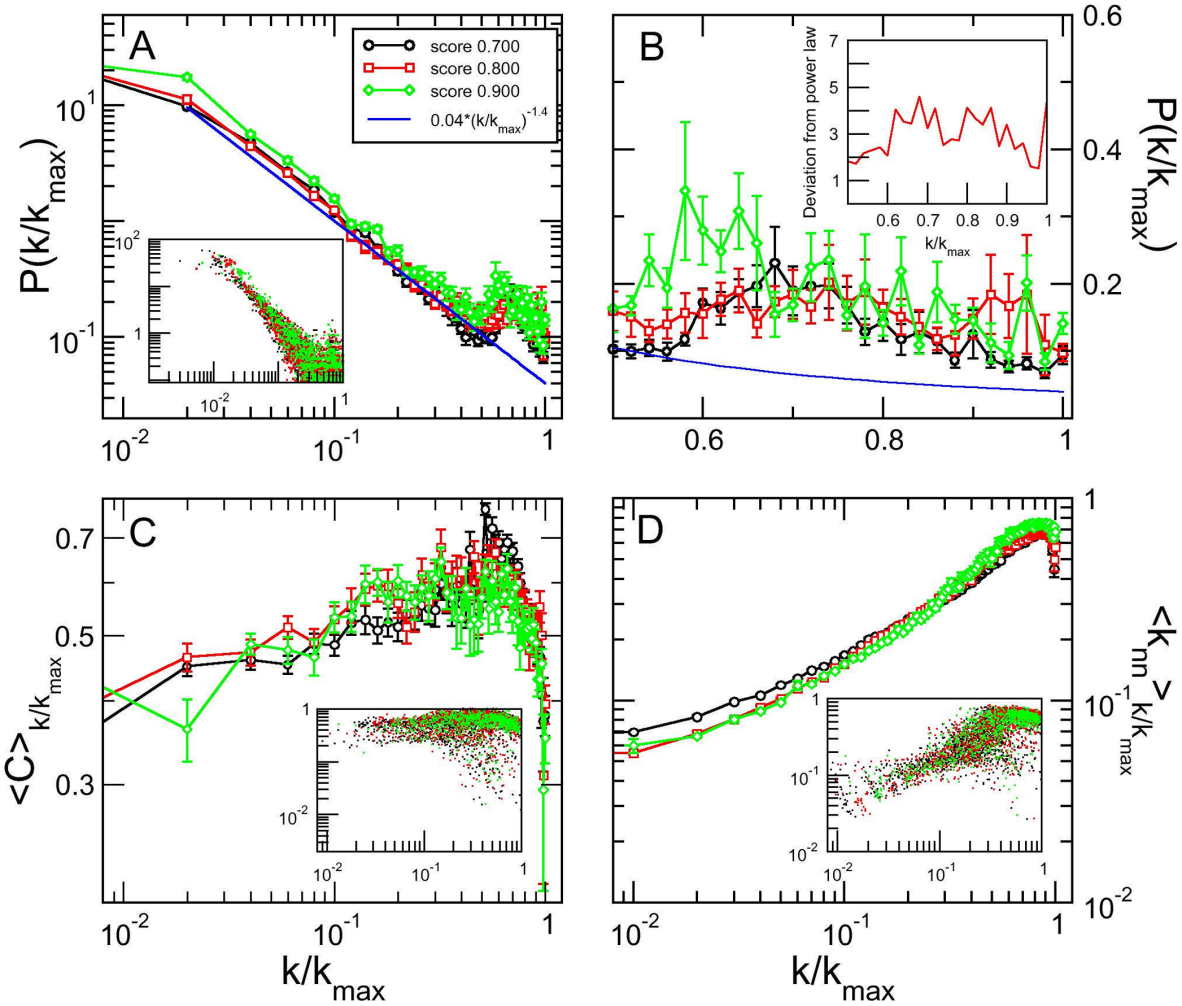
Topological quantities for all 31 core eukaryote organisms from STRING database. Three different confidence scores: 0.700, 0.800 and 0.900 (black, red and green lines in all graphs, respectively). All measurements are taken as functions of node degree rescaled by the maximum degree of the corresponding network, 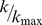. All averages were taken over intervals 
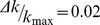
. (**a**) Average degree distribution compared with a tentative power law fit (blue line). (**b**) Average degree distribution in linear scale, showing the increase in the degree distribution for higher degree. The inset presents the distance between the power law fit and the average of networks with score 0.800 measured in number of standard deviations. (**c)** Clustering coefficient and (**d)** mean nearest neighbor degree averaged over all core organisms. The insets in panels (**a**), (**c**), and (**d**) show individual results for all 31 core organisms for each score.

Figure 1a presents the average, taken at intervals of 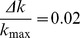, of the network degree distribution, 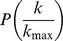 versus 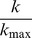 for three different confidence scores: 0.700, 0.800 and 0.900. The inset presents the degree distributions of all 31 core eukaryote organisms, with different colors for different scores. The blue line in Fig. 1a is a power law fit, 
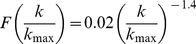
, which describes 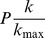 for initial values of 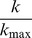. At values of 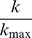 near 0.7, this degree distribution presents a local maximum, associated to the cloud of points with higher values of probability presented in the inset. The probability of proteins with degree near *k*_max_ increases and indicates a genome evolution dynamics where high degree genes are probable to appear. As the main mechanism of genome evolution is gene duplication [3], [4], it is reasonable to assume that the local maximum in 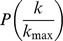 for large 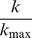 is due to high duplication probability for more connected genes. Figure 1b presents the same data in a linear plot, where the standard deviations for each average value of 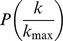 are shown, to evince that deviations from the power law fit is significant. Each point is an average over 31 core eukaryote organisms, justifying a Z test for significance. The difference between the power law fit and the average 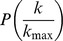 for confidence score 0.800 is shown in the inset for Fig. 1b, in units of standard deviations for 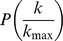, calculated at intervals of 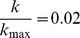. The maximum in degree distribution is significantly different from the power law. This is a novel result which has been evinced by plotting the distributions as functions of 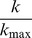, instead of functions of 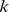 or 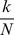. From now on, we shall refer to 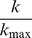 as the relative degree of a node, which varies in the interval (0,1).

Figure 1c plots, as a function of 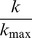,the average clustering coefficient 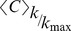, defined as the fraction of existing connections between the neighbors of a gene with *k* neighbors in relation to the maximum number of such connections 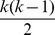. The inset in Fig. 1c individually shows the corresponding data for all core organisms. For all three scores this curve is initially constant, presenting local minimum and maximum for, roughly, 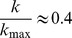 and 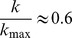, respectively, decreasing after that: the most connected genes are not the maximally clustered. Observe that, while the maximum in 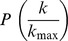 occurs for 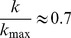, the maximum for the clustering coefficient occurs before that.

Figure 1d plots the average relative degree of the neighbors 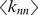 of a gene as a function of 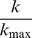. The inset individually shows the corresponding data for all core organisms. For all scores this curve is initially increasing, presenting a local maximum at roughly 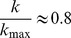, decreasing after that. It means that the most connected genes are not connected only to the most connected genes.

Summarizing, these plots indicate that *i)* 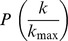 does not follow a power law; *ii)* it presents a local maximum for 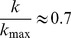; *iii)* the clustering coefficient is not uniform, presenting a local minimum and maximum; and *iv)* the network is assortative up to 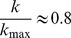, with 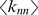 decreasing after that. These observations suggest modules of high average degree which are highly clustered. This behavior is evinced by the superposition of data from a large number of organisms, plotted against a normalized degree 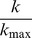. For comparison, in Text S2 we present plots where the degree *k* is normalized by the total number of genes of each organism: there this behavior is not as clearly unveiled.

We have also considered other databases. However, STRING agglutinates information from these other databases, with the further advantage of a confidence scoring. In Text S3 we explicitly present and discuss the same results for BIOGRID [15] and IRefweb [16], where we have also simulated a confidence scoring by neglecting all information in these databases that were scored as low confidence in STRING database: in these cases the results are the same as using STRING. In fact, compared to other databases, high confidence scoring in STRING is generally more stringent in what regards assigning protein-protein association based on techniques prone to false positives as high-throughput experiments.

There is another relevant aspect for genome evolution: Duplication events can be assessed by analyzing gene families, *i.e.,* genes sharing the same ancestral gene. Some gene families have mainly orthologs, while others are composed by a great number of paralogs, indicating many duplication episodes [7], [17]. The reason why some genes are prone to duplicate while others avoid duplication is controversial. However, duplication is clearly not randomly fixed and functional characteristics of the parent gene certainly influence new born genes fates. It has been discussed that genes presenting substrate promiscuity are prone to fix duplication while other genes avoid duplication because it probably leads to deleterious effects [18].

Protein-protein interaction networks properties have been used as evidence for genes with increased duplication probability. In particular, Prachumwat and Li have obtained a negative correlation between duplication and connectivity in *Saccharomyces cerevisiae* [19]. However, connectivity *per se* is somewhat ambiguous, since there are at least two classes of hub genes: (*i*) hubs taking part of biological modules, called *intramodular hubs*; and (*ii*) hubs which connect biological modules, called *intermodular hubs*. Intramodular hubs connect to several proteins which are highly connected among themselves and are jointly performing some biological task [20]. These hubs are hardly ever pleiotropic. On the other hand, intermodular hubs are generally pleiotropic and connect to different biological modules, interacting with different partners in different moments and/or different cellular compartments [21], [22]. Mathematically these two kinds of hubs may be differentiated by using two node indices: connectivity and clustering coefficient. The first merely counts the number of interacting nodes with a given node. The second index measures the level of interactions between the neighbors of a given node.

Li and collaborators [23] demonstrated that highly connected proteins with low clustering coefficient (intermodular hubs) are more probable to stem from duplicated genes as compared with proteins that are highly connected and highly clustered (intramodular hubs). According to these authors, intramodular hubs represent the network most stable and conservative part, while intermodular hubs represent evolutionary dynamic network regions with a high duplication rate. Similar results have been found by Fraser [21].

### Genome Evolution Model

These characteristics of genomes may be numerically simulated by an evolution dynamics with two different gene acquisition mechanisms: *de novo* formation and duplication. The first mechanism follows Barabási preferential attachment rule, which simulates an enhanced attachment probability shown by genes with more active domains. The second mechanism describes the facts discussed above: genes are chosen with higher probability when they are more connected, but less clustered. Protein-protein association information may be organized as a binary matrix whose elements are noted by 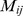, such that 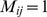 in case proteins labeled by indices *i* and *j* are associated and 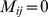 otherwise. Now, the clustering coefficient 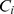 for the 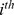 gene is defined as [24], [25]

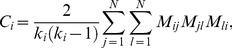
(4)which gives the ratio of existing links between the neighbors of the gene *i* to the maximum possible number of such links (which is equal to the number of combinations of 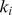 elements 2 by 2).

The duplication probability for the 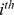 gene is defined as
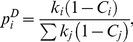
(5)where the denominator guarantees a normalized probability. This assumption reproduces the features shown by organisms protein-protein networks that *i)* degree distributions have a local maximum for 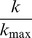 near 1 (Fig. 1) and *ii)* more clustered genes are less prone to duplicate [7], [22], [26]. For illustration, in Text S4 we presents the average value of duplication probability versus the average evolutionary plasticity index (EPI) [7] for different metabolic pathways or gene families: there is a defined positive correlation between EPI and duplication probability as given by Eq.(5). Here we remark that the network properties as clustering coefficient or degree for each node must be obtained from the whole network, comprising all genes and protein-protein association. Genes that are intermodular hubs in the whole network may become intramodular when considering a partial network. Natural selection acts over the whole organism, consequently, when correlating network properties and evolutionary plasticity, one should consider all nodes and links. So, gene families as RNA binding proteins (RBP) [20], for example, must be considered as a part of the whole genome, that is, the links with other proteins must also be considered when calculating their network properties.

Simulations start with 5 nodes, each linked to two others, forming a ring. To acquire a new gene we first choose either *de novo* mechanism, with probability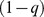, or duplication, with probability 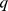. If the *de novo* mechanism is chosen, each existing node 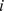 is linked to the new one with probability 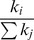, and the procedure is repeated until the new node presents at least one link. In case of duplication, the node to be duplicated is chosen by using the probability defined in Eq.(5). Duplication implies creating a new node linked to its parent and with the same neighbors.

After duplication, mutations are implemented by deleting links between either the parent or the child with a common neighbor with probability 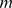. In fact, a hallmark of gene duplication is the subsequent speciation of at least one gene copy [27].

**Figure 2:**
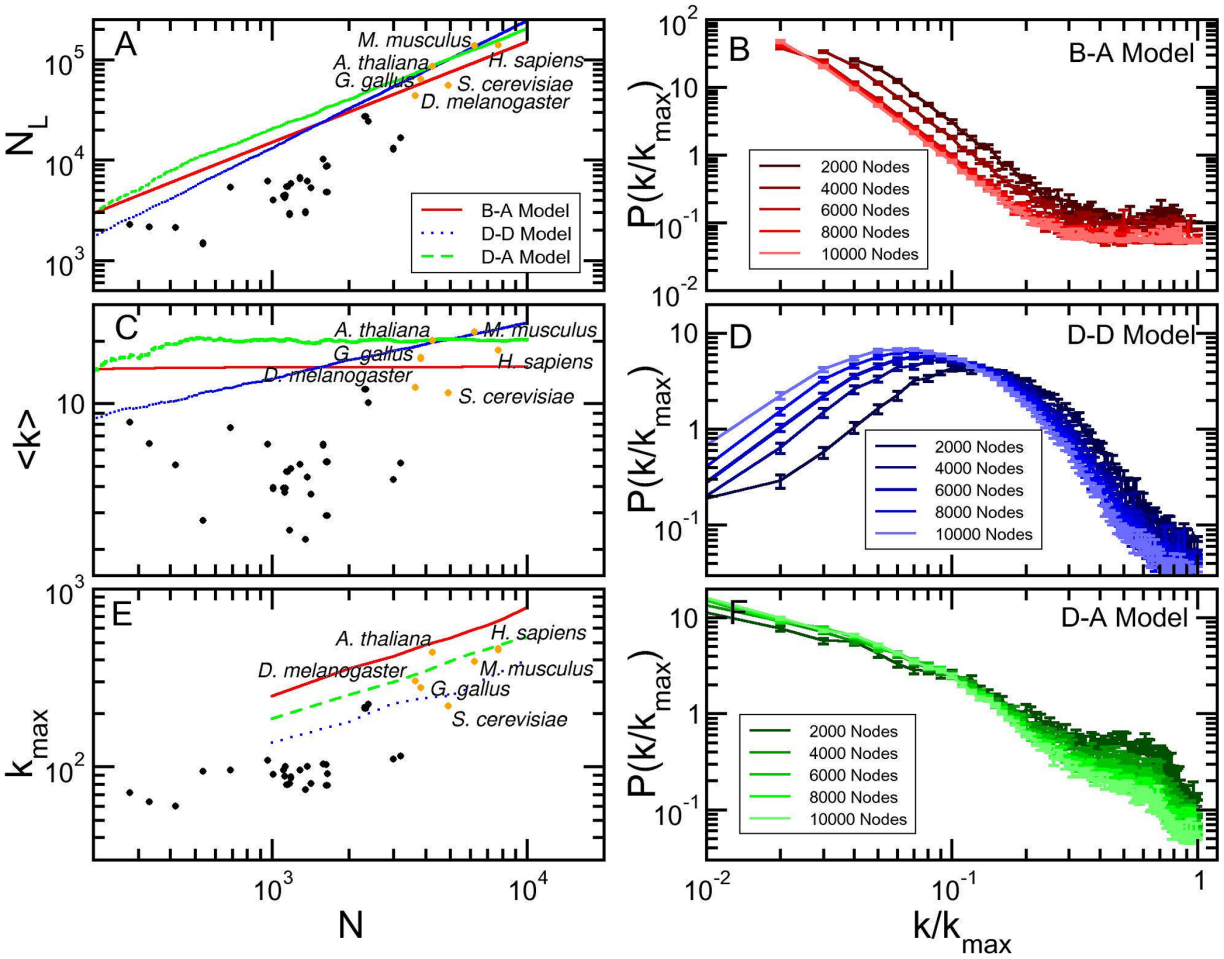
Evolution of simulated models. Barabási-Albert, duplication-divergence and duplication-acquisiton networks (red, blue and green lines, respectively). The black dots represent all core organisms from STRING database, where six well studied organisms are highlighted in orange. (a) Number of links, (c) mean degree and (e) maximum degree are shown as functions of the total number of nodes in the network. The degree distribution was calculated in five snapshots of the evolution of (b) Barabási-Albert, (d) duplication-divergence, and (f) duplication-acquisition models, in intervals of 2000 nodes.

To compare with simulated genome evolution dynamics we chose those organisms for which there is more information regarding protein-protein association. Figure 2a shows the number of links versus the number of genes for the 31 core eukaryote organisms for 0.800 confidence score. Observe that data for very well studied organisms present larger numbers of genes and links, that is, more information is available. In what follows we considered 6 organisms, marked with orange dots in Fig. 2a (*Homo sapiens*, *Mus musculus*, *Arabidopsis thaliana, Drosophila melanogaster, Saccharomyces cerevisiae,* and *Gallus gallus).*

The present simulation model has two parameters, duplication probability 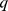 and mutation probability 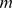. For the numbers of links and genes of simulated networks to fall in the same intervals as more extensively investigated organisms (Fig. 2a), 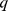
must be of the order of 0.90, which is experimentally verified: Zhou *et al*. [4] have studied *Drosophila melanogaster* genome and compared to other organisms in *D. melanogaster* subgroup. They have found that duplication is responsible for 80% of new genes, and 10% is generated by retroposition, here taken as an additional form of gene duplication. We are left with one single parameter, 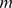, set to 0.05 to match the observed relation between number of links and nodes presented by protein-protein association matrices of real organisms (Fig. 2a). Results for different values of these parameters are discussed in Text S5.

We also simulated two other well described models for genome evolution: Barabási and Albert model [1], based on a preferential attachment rule, and Vazquez *et al.* [5], [6] model, where genomes are built by duplicating randomly chosen genes. For both models, parameters are set to ensure that the number of links and nodes are roughly the same as in the protein-protein association networks obtained from STRING database for confidence score 0.800 (For other parameter values, see Text S5). In Barabási-Albert model, each new node is connected with 15 neighbors, and in the duplication-divergence model each node is linked with its parent, and has 0.4 of mutation probability. For brevity, we considered the most cited models in the literature although other interesting models also address genome growth [28]–[31].

Figs. 2a, 2c, and 2e present, as a function of 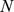, the plots of number of links 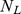, average degree 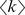, and maximum degree, 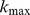, for experimental results (dots) and simulated models (solid lines). As discussed, the chosen model parameters ensure that the simulated number of links crosses the region with best investigated organisms (orange dots). The experimental points indicate that the number of links is proportional to the number of nodes, that is, 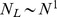. This behavior is clearly shown by both Barabási-Albert and our model, and is further evinced by Fig. 2c, that shows a constant average degree for experimental dots and these two models. Finally, Fig. 2e shows that, for the simulations, 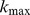 increases with, roughly, 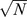. The results for organisms are not in contradiction, although they are not conclusive. Anyway, this behavior explains why using 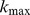
instead of 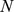 as the normalization constant in Eq. 2 yields different results.

Figs. 2b, 2d, and 2f present 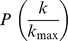 versus 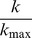 for the three simulated models, measured in networks of different sizes. Observe that clearly Barabási-Albert and our model converge to a scaling invariant distributions that superpose as 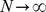, while for Vázquez (D–D) model this convergence is either not true or too slow. This is a relevant point: although real genomes are finite, we may speculate that when large enough they present a scale invariant degree distribution. If this is true, the data collapse predicted by scaling invariance, together with a significant fit of the collapsed degree distribution of all core organisms, is as a strong evidence of a common mechanism universally ruling eukaryotes genome growth.

On the other hand, degree distributions for real organisms may present finite size effects. For example, both STRING data and D–A model results show that smaller networks present a higher local maximum in 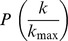 for large 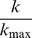. To properly compare the simulations results with experimental networks with variable sizes, we considered a weighted average of the degree distribution, as follows.

**Figure 3:**
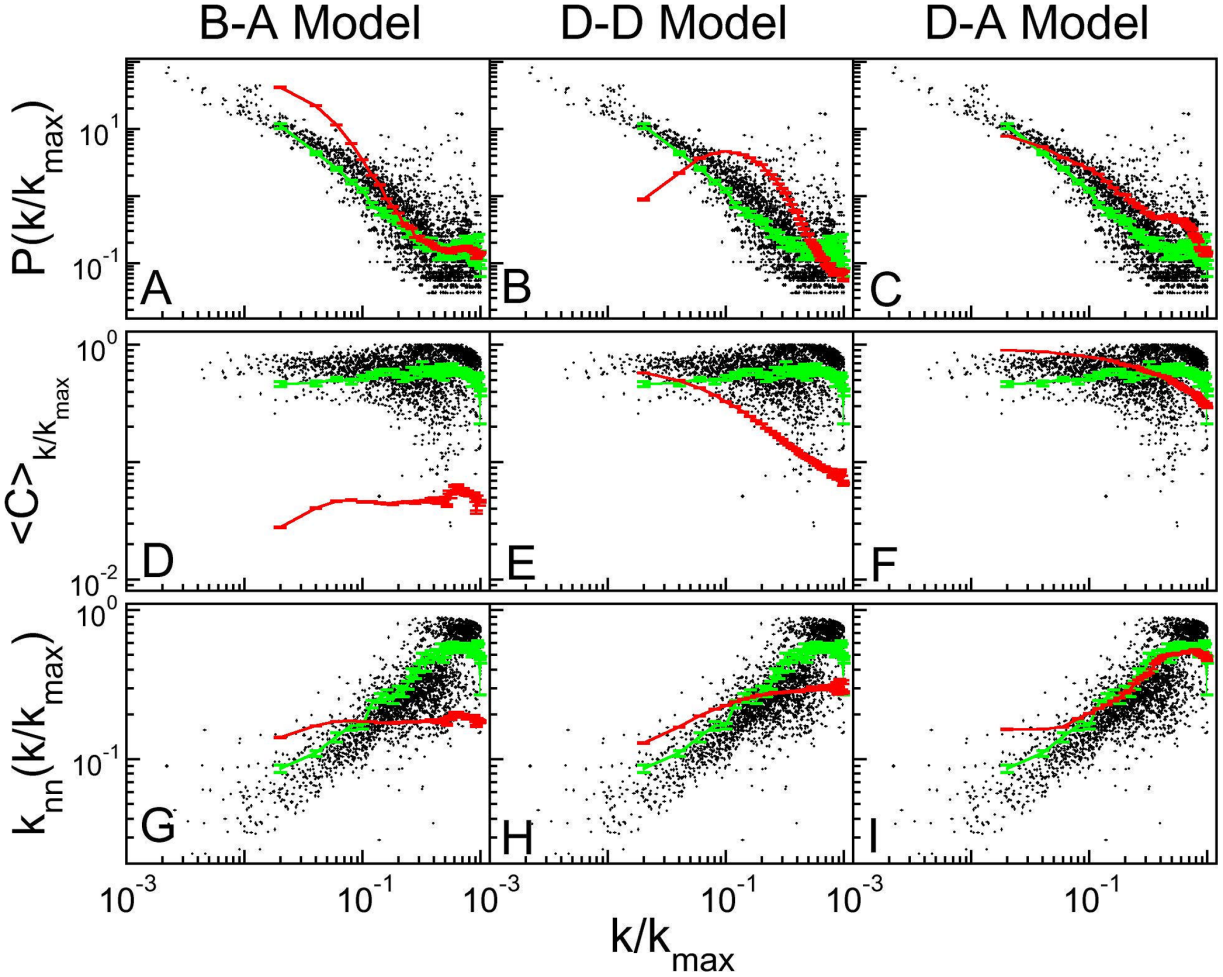
Comparison of topological measures for simulated networks. The black dots represent the superposed networks for all core organisms from string database with confidence score 0.800, the green lines are averages taken in intervals of 
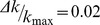
, and the red lines are weighted averages of simulated networks. The upper, central, and lower rows show, respectively, degree distribution, clustering coefficient, and nearest neighbor mean degree. Each column refers to a simulated model: Barabási-Albert on the left, duplication-divergence on the center and duplication-acquisition on the right.

For each model, we produced 10 samples in each size range, with size ranges being 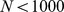, 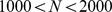, …, 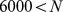, and obtained the distributions of degree, clustering coefficient, and average degree of the neighbors as functions of 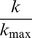. To compare with the set of all 31 core eukaryote organisms, presenting, respectively, 6, 15, 2, 4, 2, 0 and 2 organisms in each size range, we produced weighted averages over the size ranges for the topological distributions, using the weights 6/31, 15/31, 2/31, 4/31, 2/31, 0/31 and 2/31. These results are shown in Fig. 3.

Other parameters values in each model yield different results, as discussed in Text S5: the description of topological quantities are worse in these cases. Similar averages for the six, best investigated organisms are shown in Figure S1.

### Duplication-acquisition Model Reproduces the Topology of Protein-protein Association Networks

For each network, we calculated the weighted average for probability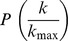, the clustering coefficient 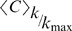, and the relative degree 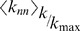
of the neighbors of a node with degree, defined as
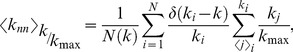
(6)where 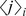 stands for a sum over the nodes 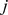 that are neighbors to node *i*, and 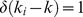 if 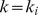 and 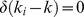 otherwise.

The black dots in Fig. 3 refer to protein-protein association networks of the 31 core eukaryote organisms. All plots indicate large clustering coefficients for all degrees, decreasing as 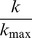 approaches 1: very high degree nodes are less clustered than less connected nodes. In organisms, the average number of connections of the neighbors, 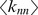, first increases with the node degree and then decreases, reinforcing the fact of very high degree nodes not presenting the largest clustering coefficient. Figure 3 presents three columns, one for each model, where we show *i)* raw data for the 31 eukaryotes as black points, weighted averages for *ii)* the 31 eukaryotes as green lines and for *iii)* simulation as red lines. The first column shows that B–A model produces a degree distribution that follows a power law, a clustering coefficient that is roughly constant at a value much less than those shown by the organisms data. Furthermore, 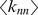 does not depend on 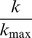. The deviation from the STRING dots reflects that Barabási-Albert model yield scale free networks with a global central hub.

The second column presents the results for the Duplication-Divergence (D–D) model. Here, this distribution clearly does not follow a power law, due to the chosen parameters (link deleting probability of 0.4), that fixed the ratio of number of links to number of nodes at the desired values (see Fig. 2a). The average clustering coefficient decreases too abruptly, as compared to organisms data: as degree increases, the clustering decreases as 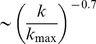. However, the average degree of the neighbors presents a mild increase, meaning that genes connect to groups of genes with slightly larger degrees.

The third column in Fig. 3 refers to the results of our model. In Fig. 3c, 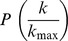
describes very well the data from STRING. For high values of 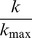, degree distribution reproduces the local maximum as shown by real organisms, although for smaller degrees. The clustering coefficient, shown in Fig. 3f, describes the major part of the interval, presenting a more intense decrease as 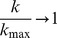. The varying character of assortativeness as 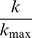 increases is also evident in Fig. 3i: 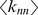 first increases to a maximum up to 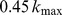.

Comparing the three columns we conclude that D–A model better catches the topological properties of protein-protein association networks, according to the currently available data in STRING, although the description is not perfect.

### Global Aspect of Protein-protein Association Matrix

Furthermore, to evince global properties of the networks, the protein-protein association data that is organized on the matrix 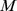 where each axis represents the protein list in a given order. The matrix elements 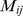 are assigned with value 1 (0) if there is (not) an association between the genes at positions *i* and *j* of the list. For illustrational purposes, these association matrices may be represented by plots where a black dot at position 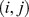 indicates that 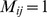.

We obtain the sets of genes of each organism from STRING database and dispose them in randomly ordered lists. Each possible order for a gene list implies a different configuration for matrix 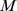, for which a cost function *E* may be defined as
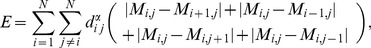
(7)where 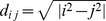 is proportional to the distance on the matrix from the point 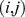 to the diagonal (when 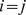), and 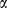 is a parameter, here taken 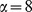. Minimization of this function, by changing the genes localization on the list, implies approximating mutually interacting genes, as discussed by Rybarczyk-Filho *et al*. [32].

The ordering algorithm starts from a randomly ordered matrix configuration and proceeds by randomly choosing a pair of genes whose positions are tentatively swapped. The cost function for this changed configuration is calculated and, in case the cost decreases, the change is accepted. If the cost function increases by 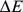, the change is accepted with probability 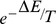, where 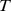 is a parameter. This procedure is intended to avoid metastable states in the optimization of Eq.(3). Finally, when 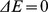, the change is accepted with 50% probability. The algorithm proceeds by randomly choosing another pair of genes and the procedure is repeated until the value of the cost function is stabilized.

**Figure 4:**
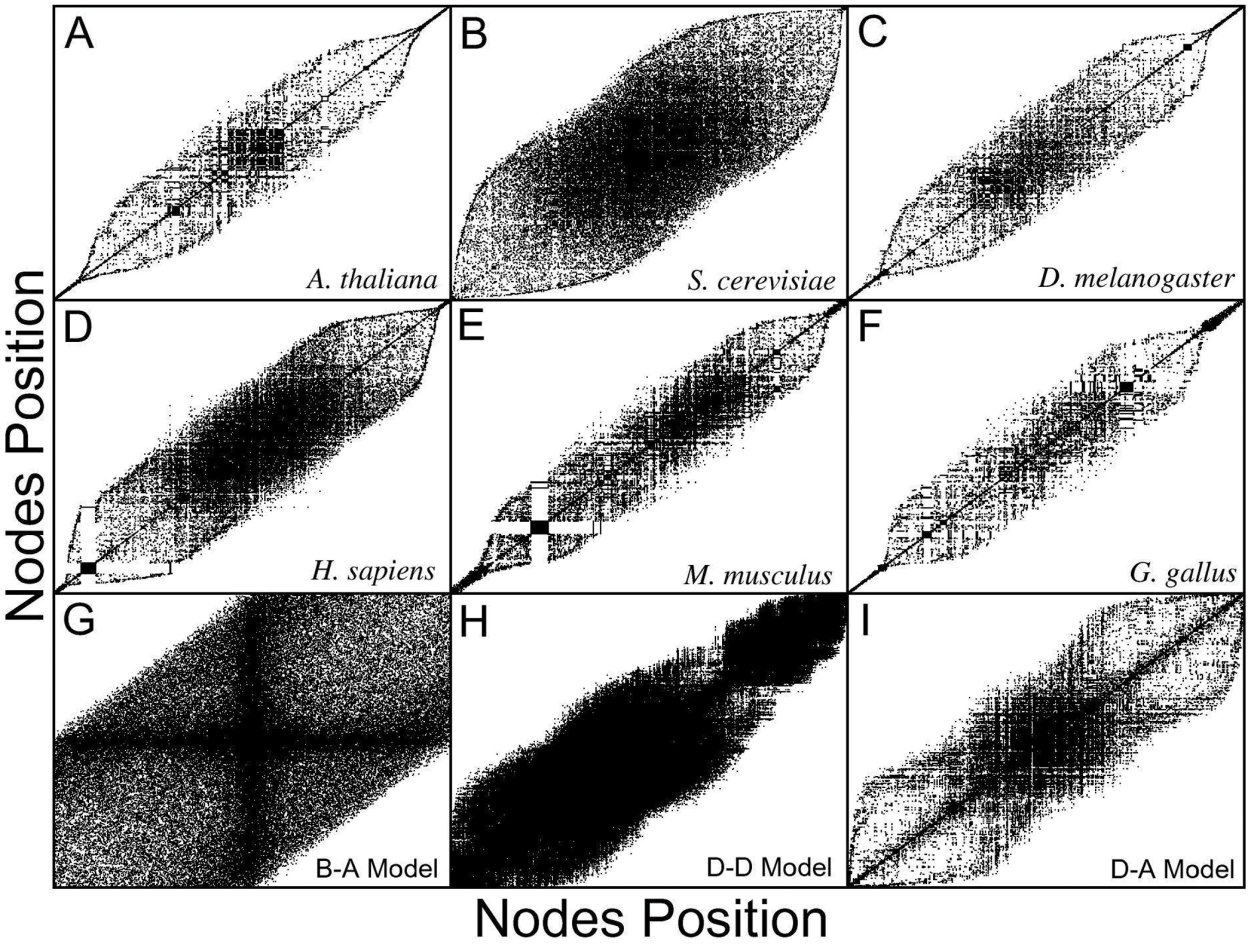
Ordered association matrices. This figure presents the association matrices for *Homo sapiens*, *Mus musculus*, *Arabidopsis thaliana, Drosophila melanogaster, Saccharomyces cerevisiae, Gallus gallus*, Barbási-Albert model, duplication-divergence model and duplication-acquisition model after running the ordering algorithm. The black dots represent protein-protein association between two nodes.

Randomly ordered lists yield association matrix configurations with black dots spread over the whole plot. Ordering the gene list by minimizing the cost function evinces topological properties of protein-protein association networks. Figure 4a–f presents the ordered matrices for the six organisms listed above. Observe that points concentrate near the diagonal, implying that there may be an association (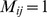) between the products of genes localized at not far apart positions *i* and *j*. Not all networks may be put in formats like those shown by Figs. 4a–f. See Fig. 4-g which represents a network built using Barabási-Albert algorithm, or an Erdös-Rényi network, presented on Text S6. In fact, this format reveals that genomes (Figs. 4a–f) do not present one central hub linked to the whole network (which could indicate scale free networks) but, contrarily, present many hubs with neighborhoods that do not span the entire system.

Figures 4g–i present ordered association matrices for simulated networks. Barabási-Albert (B–A) model (Fig. 4g) clearly shows only one module, with a central hub connected to all network. Duplication-Divergence (D–D) model, on the other hand, shows a slimmer structure around the diagonal, and Duplication-Acquisition (D–A) model presents a central hub not connected to the whole network. The ordering algorithm is further discussed in Text S6, where the same panels as Figure 4 are presented, but zooming at the central regions: the hierarchical structure of clusters, evinced by small solid squares, is clearly present in organisms and Duplication-Acquisition model. In Text S6 we also present the orderings obtained with 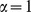, which stresses further the clustered structures.

Together, figures 1 and 4 evince different aspects of real genomes. First, degree distribution is not a power law. Second, there is an accumulation of high degree nodes, which may be explained by an enhanced duplication probability for highly connected gene products. Finally, hub genes are not central to the whole network, which presents hierarchical clusters.

## Discussion and Conclusions

In this paper we have presented evidence obtained from protein-protein association data that degree distribution is not scale free, presenting an increased probability for high degree nodes, and that there are a few hub nodes in these networks, probably organized in a hierarchical way. Furthermore, when scaled by the maximum degree in each network, 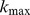, the degree distribution seems to approach a scale invariant state as the number of genes in the network increases. However, real genomes still present finite size effects. This scenario indicates a universal mechanism for genome evolution.

The understanding of genome growth mechanisms is a central point in evolutionary biology. It is well established that gene duplication is the main process for new genes emergence. Therefore, it is reasonable to think that gene duplication represents an essential feature for genome evolution. This idea has been used by Vázquez in his genome evolution model including gene duplication as genetic novelty source [5], [6]. However, in that model, genes are randomly chosen to duplicate whereas experimental evidence indicates that gene duplication is not random. There are huge differences in the fixation probability of a gene duplication event. Depending on gene niche, the new copy could be selectively fixed or eliminated [18]. This concept becomes clear when gene families are assessed. There are some gene families composed basically by vertically inherence (*i.e.* orthologs), without duplication episodes. On the other hand, there are gene families composed by great number of duplication-generated genes (*i.e.* paralogs) [7], [17]. The question is what gene characteristics will increase the fixation probability of its duplication?

The local maximum shown in Figure 1a gives us a clue about gene duplication dynamics. According to the figure, there is an increased probability of very connected proteins, indicating a genome evolution dynamics favoring hub genes emergence. However, as discussed previously, there are at least two very distinct classes of hub genes: (*i)* intramodular hubs, presenting high degree and high clustering coefficient, and (*ii*) intermodular hubs, presenting high degree and low clustering coefficient. The first one takes part in modules, which generally comprises intricate biological systems where all proteins exercise coordinate functions. In many of those systems, stoichiometry relationship is needed and a duplication event could be deleterious to the whole system. The second connects different modules, commonly exercising pleiotropic functions. Gene duplication theories always associate the fixation of the new-born gene copy with new function development [27]. Additionally, a gene performing more than one function - when each function cannot be independently optimized - could benefit from a duplication event where each gene copy is rendered free to independently optimize different functions [33].

Intermodular hubs have been discussed as targets of gene duplication [23]. This feature can be explained by the catalytic versatility of intermodular hubs [34]. These promiscuous activities often serve as starting points for the evolution of new functions if, or when, necessary [35]. Example of intermodular hubs, members of the Per-Arnt-Sim (PAS) receptor family recognize a huge variability of ligands, from photons to polyaromatic hydrocarbons. This receptor family presents 34 proteins in mammals and thousand proteins among genomes of many other species, evincing lots of duplication episodes [36]. Also, Szklarczyk *et al.* have shown that for yeast in nearly 70% of small scale duplication events, the paralogs do not remain working in the same complex and in at least 40% their ancestor gene should participate in more than one biological module [37].

On the other hand, intramodular hubs are associated to ancient networks that have reached their architecture early in evolution and any modification can affect their homeostasis [7]. This fact is well exemplified by ribosomes and DNA repair mechanisms, both very ancient systems with modular network architecture and both composed by genes with almost none duplication episode fixed though their evolutionary history [7], [20].

Finally, a clear positive correlation between the network quantity 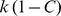 averaged over gene families and the average evolutionary plasticity index as discussed in Text S4 further supports the idea of intermodular hubs as preferential gene duplication targets in comparison to intramodular hubs.

Here, we propose a simulation model for genome evolution, Duplication-Acquisition model, where genes in a network are either duplicated or acquired *de novo* using a preferential attachment rule. However, according to our model, genes are not arbitrarily chosen to duplicate: the duplication probability linearly grows with gene degree and decreases with its clustering coefficient. In other words, intermodular hubs have increased probability to duplicate. With this simple rule, topological distributions of biological networks are well described. This model correctly predicts that, to produce protein-protein association networks with number of links and number of nodes in the observed range for eukaryotes, it is necessary 90% of gene duplication and 10% of *de novo* gene acquisition.

A final remark on the model contemplates whole genome duplication, which is not explicitly taken into account. However, such a duplication in the first moment would not change the results, since each gene would be connected to twice the number of other nodes and, as also 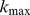 duplicates, the relative degree of each node, 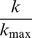 remains the same. In a second moment, there could be more room for evolution, but in this case, the fixation probability would follow the same reasoning as in the original model. So, we do not expect that whole genome duplication would greatly change the genome topological distributions as compared to our model.

To compare the networks we ordered gene lists for each organism and model to produce protein-protein association matrices yielding images of the network association structure. These images give a global assessment of the networks, suggesting that there is a system scale that is less than its size (see Fig. 4), with, possibly, a hierarchical modular organization, as predicted by the Duplication-Acquisition model (see Text S6).

The simulation model is not perfect. Phenotypic effects caused by gene acquisition, duplication, or mutation cannot be fully grasped by network gene properties only and, consequently, this model is an over-simplification. However it does point towards a positive correlation between duplication probability and degree, while indicating a negative correlation between duplication probability and clustering coefficient. Consequently, Duplication-Acquisition model suggest how and where evolution works to build genetic novelty.

## Supporting Information

Figure S1
**Comparison of topological measures for the simulated networks.** Black dots represent the superposed networks for six organisms from STRING database with confidence score 0.800 (*Homo sapiens*, *Mus musculus*, *Arabidopsis thaliana, Drosophila melanogaster, Saccharomyces cerevisiae,* and *Gallus gallus)*, red lines are averages of these networks taken in intervals 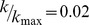, and green lines are weighted averages of simulated networks. Upper, central, and lower rows show, respectively, degree distribution, clustering coefficient, and nearest neighbor mean degree. Each column refers to a simulated model: Barabási-Albert on the left, duplication-divergence on the center and duplication-acquisition on the right.(TIFF)

Text S1 **STRING confidence score.** In this text we discuss the choice of STRING confidence score and its effect in the number of links and nodes for six organisms networks.
(PDF)

Text S2 **Degree normalization.** In this text we discuss the effect of a different normalization when comparing several different networks, and how the adequate normalization can evince topological properties.
(PDF)

Text S3 **Comparison with other databases.** STRING database is compared with BioGRID an iRefWeb databases.
(PDF)

Text S4 **Correlation between network properties and Evolutionary Plasticity Index (EPI).** In this text we present and discuss a plot of the average duplication probability for different gene families versus the average Evolutionary Plasticity Index (EPI), in order to further support the model assumption of a duplication probability.
(PDF)

Text S5 **Parameters of the models.** In this text we discuss the choice of parameters in Barabási-Albert, Duplication-Divergence, and Duplication-Acquisition models.
(PDF)

Text S6 **Ordering Algorithm.** In this text we further discuss the ordering algorithm, and present some properties of networks that can be evinced with this algorithm.
(PDF)
