# Correction: Preferential Duplication of Intermodular Hub Genes: An Evolutionary Signature in Eukaryotes Genome Networks

**DOI:** 10.1371/journal.pone.0118425

**Published:** 2015-03-13

**Authors:** Ricardo M. Ferreira, José Luiz Rybarczyk-Filho, Rodrigo J. S. Dalmolin, Mauro A. A. Castro, José C. F. Moreira, Leonardo G. Brunnet, Rita M. C. de Almeida

We have spotted an important error in our paper. The probability for gene duplication actually used in our simulations is inversely proportional to the node degree, instead of the one given by equation (5) presented in the paper, where this probability appears as directly proportional.

Although the conclusions remain valid in what regards the typical clusterization of the duplicated genes, and that highly clustered gene present a very small duplication probability, one important consequence is that the duplicated genes are not the hubs, but mostly those with small degrees.

It remains true that among hubs, intermodular ones are more prone to duplication in comparison to highly clustered hubs. However, a duplication probability that is inversely proportional to degree implies that, for genes with the same clustering coefficient, those with less neighbors have a higher chance to duplicate, contrarily to what is concluded in the paper.

This error does not affect the conclusions regarding the data analysis presented in Fig. 1.
