# Supplementary figures and images for: Preferential Duplication of Intermodular Hub Genes: An Evolutionary Signature in Eukaryotes Genome Networks
Source: PLoS One. 2013 Feb 26;8(2):e56579. doi: 10.1371/journal.pone.0056579 (PMC3582557; doi:10.1371/journal.pone.0056579)

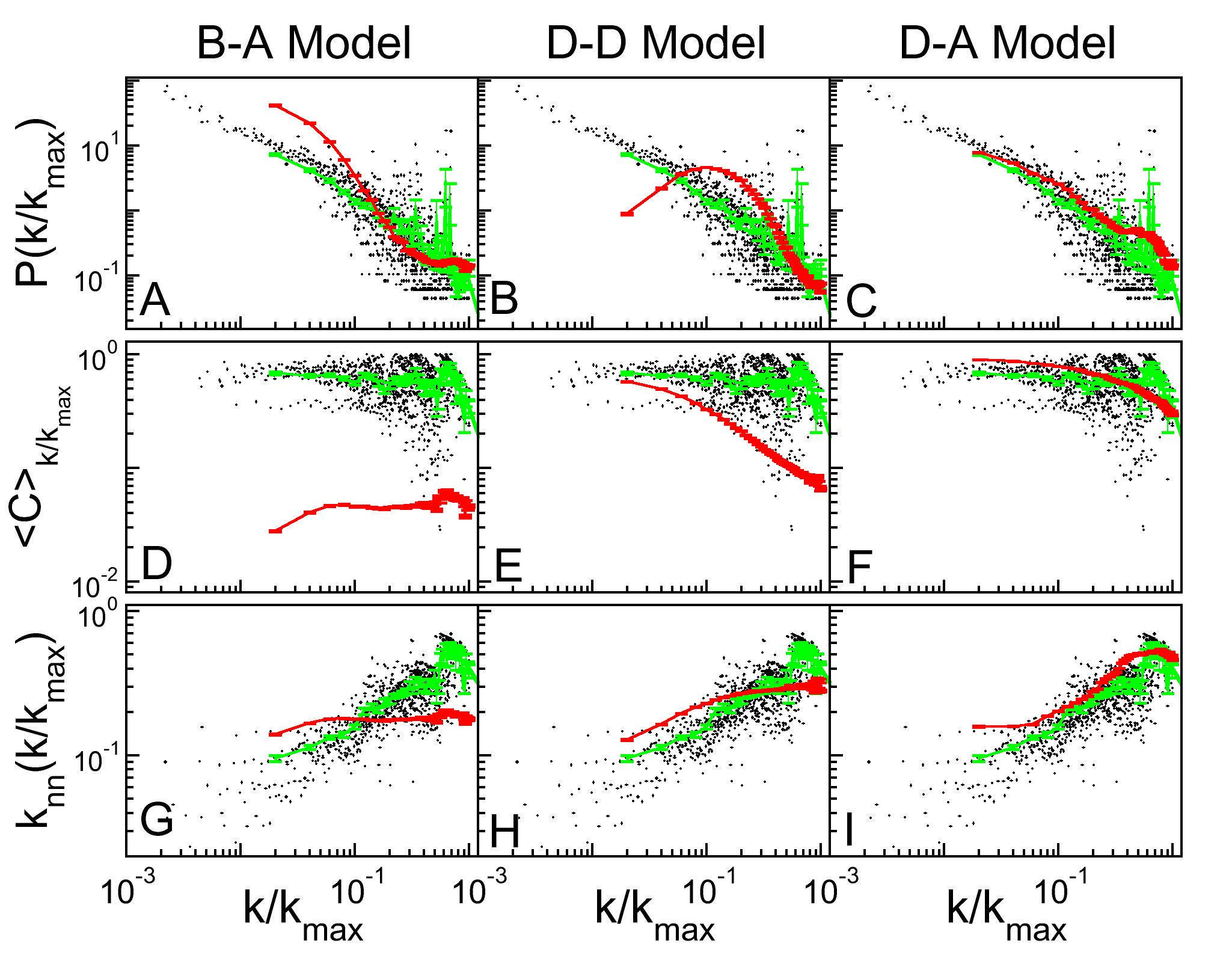

Supplement: Figure S1 — Comparison of topological measures for the simulated networks. Black dots represent the superposed networks for six organisms from STRING database with confidence score 0.800 (Homo sapiens, Mus musculus, Arabidopsis thaliana, Drosophila melanogaster, Saccharomyces cerevisiae, and Gallus gallus), red lines are averages of these networks taken in intervals , and green lines are weighted averages of simulated networks. Upper, central, and lower rows show, respectively, degree distribution, clustering coefficient, and nearest neighbor mean degree. Each column refers to a simulated model: Barabási-Albert on the left, duplication-divergence on the center and duplication-acquisition on the right. (TIFF) [file pone.0056579.s001.tif]
