# Supplementary material for: Preferential Duplication of Intermodular Hub Genes: An Evolutionary Signature in Eukaryotes Genome Networks
Source: PLoS One. 2013 Feb 26;8(2):e56579. doi: 10.1371/journal.pone.0056579 (PMC3582557; doi:10.1371/journal.pone.0056579)
Supplement: Text S1 — STRING confidence score. In this text we discuss the choice of STRING confidence score and its effect in the number of links and nodes for six organisms networks. (PDF) [file pone.0056579.s002.pdf]

Supplementary material online for

Preferential duplication of intermodular hub genes: an evolutionary signature in eukaryotes genome networks.

Ricardo M. Ferreira*[1],José Luiz Rybarczyk-Filho*[1], Rodrigo J. S. Dalmolin*[3], Mauro A. A. Castro[1,2], José C. F. Moreira[3], Leonardo G. Brunnet[1]& Rita M. C. de Almeida[1,2]

Instituto de Física[1], NationalInstituteof Science and Technology for Complex Systems[2], and Departamento de Bioquímica[3], Universidade Federal do Rio Grande do Sul, Av. Bento Gonçalves, 9500, 91051-970 C.P. 15051, Porto Alegre, Brazil.

***These authors contributed equally to this paper**

Correspondenceto:

Rita M. C. de Almeida
Instituto de Física, Universidade Federal do Rio Grande do Sul,
Av. Bento Gonçalves, 9500, 91051-970 C.P. 15051, Porto Alegre, Brazil.

STRING confidence score.

STRING database [1-3] assigns protein-protein association using different methods. It also provides a confidence score, which allows the user to tune the desired certainty that a given association is true. When dealing with a large network, a too stringent confidence score may introduce too many false negatives. To choose the adequate STRING confidence scoreto build the networks we have considered the six most studied organisms and plotted the number of links versus the number of nodes in the networks using different confidence scores. Figure 1 shows a change of behavior from score 0.800 to 0.900, with the number of links presenting a noticeable drop, which we interpreted as an indication of a too stringent score, from which too many false negatives would be introduced.

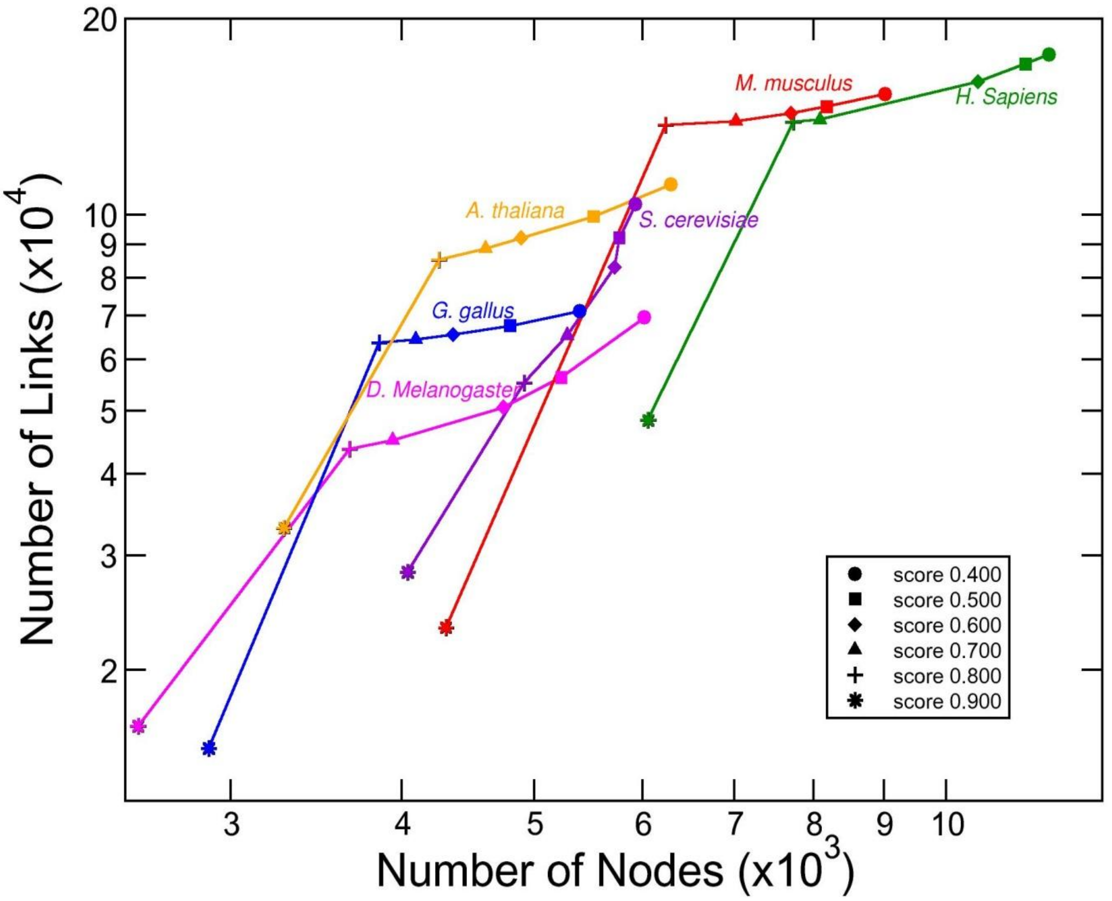

Figure 1.Number of links *versus* number of nodes for different scores. In this figure we can see the effect of confidence score in the number of links and nodes for six organisms networks.

References

1. Jensen LJ, Kuhn M, Stark M, Chaffron S, Creevey C, Muller J, Doerks T, Julien P, Roth A, Simonovic M, Bork P, von Mering C (2009) STRING 8--a global viewonproteinsandtheirfunctionalinteractions in 630 organisms. NuclAcids Res 37: D412-D416.

2. von Mering C, Jensen LJ, Snel B, Hooper SD, Krupp M, Foglierini M, Jouffre N, Huynen MA, Bork P (2005) STRING: knownandpredictedprotein-proteinassociations, integratedandtransferredacrossorganisms. NuclAcids Res 33: D433-D437.

3. von Mering C, Jensen LJ, Kuhn M, Chaffron S, Doerks T, Kruger B, Snel B, Bork P (2007) STRING 7--recentdevelopments in theintegrationandpredictionofproteininteractions. NuclAcids Res 35: D358-D362.
