# Supplementary material for: Preferential Duplication of Intermodular Hub Genes: An Evolutionary Signature in Eukaryotes Genome Networks
Source: PLoS One. 2013 Feb 26;8(2):e56579. doi: 10.1371/journal.pone.0056579 (PMC3582557; doi:10.1371/journal.pone.0056579)
Supplement: Text S2 — Degree normalization. In this text we discuss the effect of a different normalization when comparing several different networks, and how the adequate normalization can evince topological properties. (PDF) [file pone.0056579.s003.pdf]

# Supplementary material online for

## Preferential duplication of intermodular hub genes: an evolutionary signature in eukaryotes genome networks.

Ricardo M. Ferreira*[1],José Luiz Rybarczyk-Filho*[1], Rodrigo J. S. Dalmolin*[3], Mauro A. A. Castro[1,2], José C. F. Moreira[3], Leonardo G. Brunnet[1]& Rita M. C. de Almeida[1,2]

Instituto de Física[1], NationalInstituteof Science and Technology for Complex Systems[2], and Departamento de Bioquímica[3], Universidade Federal do Rio Grande do Sul, Av. Bento Gonçalves, 9500, 91051-970 C.P. 15051, Porto Alegre, Brazil.

***These authors contributed equally to this paper**

**Correspondenceto:**

Rita M. C. de Almeida
Instituto de Física, Universidade Federal do Rio Grande do Sul,
Av. Bento Gonçalves, 9500, 91051-970 C.P. 15051, Porto Alegre, Brazil.

**Degree normalization.**

In the main text we found some properties of networks are evinced by superposing a large number of networks, namely: *i)* degree distribution is not power law; *ii)* presents a local maximum for $k/k_{\max} = 0.7$; *iii)* the clustering coefficient is not uniform, presenting local minimum and maximum; and *iv)* the network is assortative up to $k/k_{\max} = 0.8$, decreasing after that. To be able to compare networks of different sizes we rescale them by its $k_{\max}$. In Figure 1we test normalization by $N$ that also allow comparison between networks of different sizes. We can see that degree distribution, clustering coefficient and mean degree of nearest neighbors do not show any local maxima. Instead, they present a monotonic behavior with a large dispersion, not allowing any conclusion about highly connected modules.

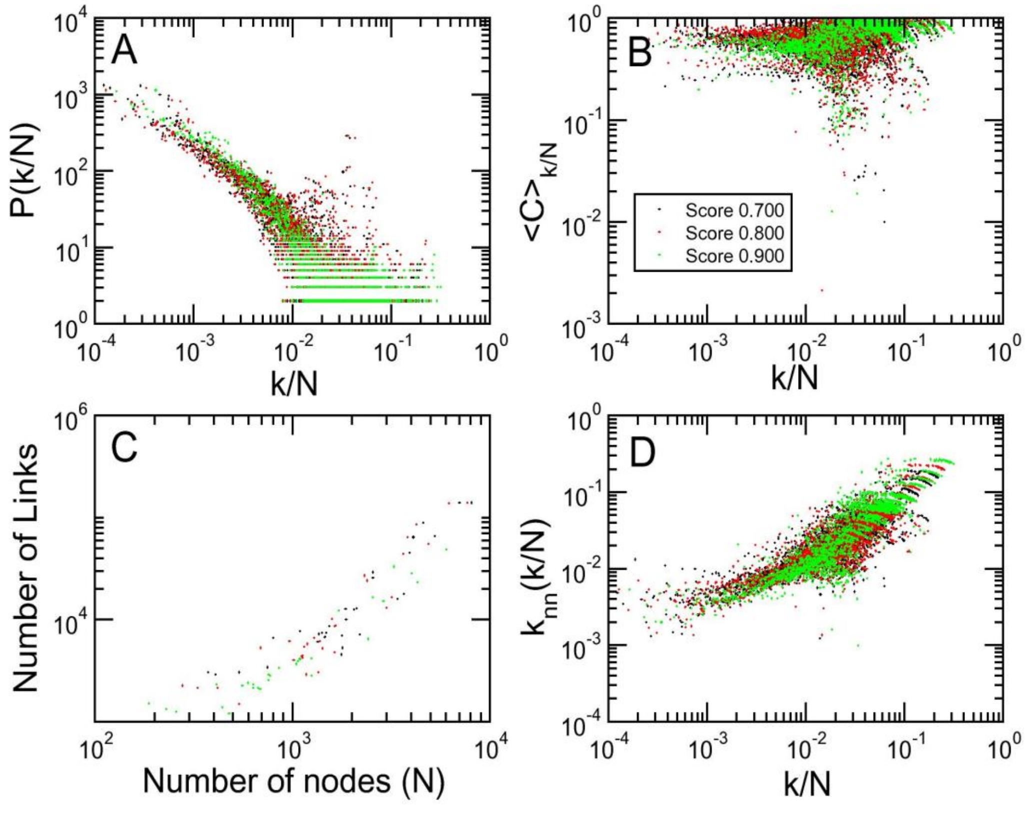


**Figure 1.Topological measures for 31 eukaryote core organisms**. STRING database with confidence scores of 0.700, 0.800, and 0.900 (respectively black, red, and green dots), with degree rescaled by number of nodes $N$. (**a**) Degree distribution, (**b**) clustering coefficient, (**c**) number of links per number of nodes, and (**d**) mean average degree of nearest neighbors. The properties discussed in the main text, such as the increase in the probability of high degree nodes, are not clearly evinced when degree $k$ is scaled by $N$.
