# Supplementary material for: Preferential Duplication of Intermodular Hub Genes: An Evolutionary Signature in Eukaryotes Genome Networks
Source: PLoS One. 2013 Feb 26;8(2):e56579. doi: 10.1371/journal.pone.0056579 (PMC3582557; doi:10.1371/journal.pone.0056579)
Supplement: Text S3 — Comparison with other databases. STRING database is compared with BioGRID an iRefWeb databases. (PDF) [file pone.0056579.s004.pdf]

Supplementary material online for

Preferential duplication of intermodular hub genes: an evolutionary signature in eukaryotes genome networks.

Ricardo M. Ferreira*[1],José Luiz Rybarczyk-Filho*[1], Rodrigo J. S. Dalmolin*[3], Mauro A. A. Castro[1,2], José C. F. Moreira[3], Leonardo G. Brunnet[1]& Rita M. C. de Almeida[1,2]

Instituto de Física[1], NationalInstituteof Science and Technology for Complex Systems[2], and Departamento de Bioquímica[3], Universidade Federal do Rio Grande do Sul, Av. Bento Gonçalves, 9500, 91051-970 C.P. 15051, Porto Alegre, Brazil.

***These authors contributed equally to this paper**

Correspondenceto:

Rita M. C. de Almeida
Instituto de Física, Universidade Federal do Rio Grande do Sul,
Av. Bento Gonçalves, 9500, 91051-970 C.P. 15051, Porto Alegre, Brazil.

Comparison with other databases

In order to assess the effect of choosing STRING database on our results we compared with networks built from other databases. We compared first with yeast networks obtained from BioGRID [1] and iRefWeb [2] in Fig. 1. These databases infer protein-protein associations from high-throughput experiments which may produce false positives, which do not occur *in vivo*. In the lack of a confidence score for these databases, we artificially built networks where we neglected all links in BioGRID and iRefWeb that were not present in STRING database score 0.800, without adding any other link. Figure 2 shows that by just removing the links with low STRING-confidence score all the networks present very similar results. Figure 1 compares the same networks to yeast network in STRING database. We can see that for score 0.400 the network is almost the same for all original databases, and the modified networks from BioGRID and iRefWeb are very similar to STRING network with score 0.800.

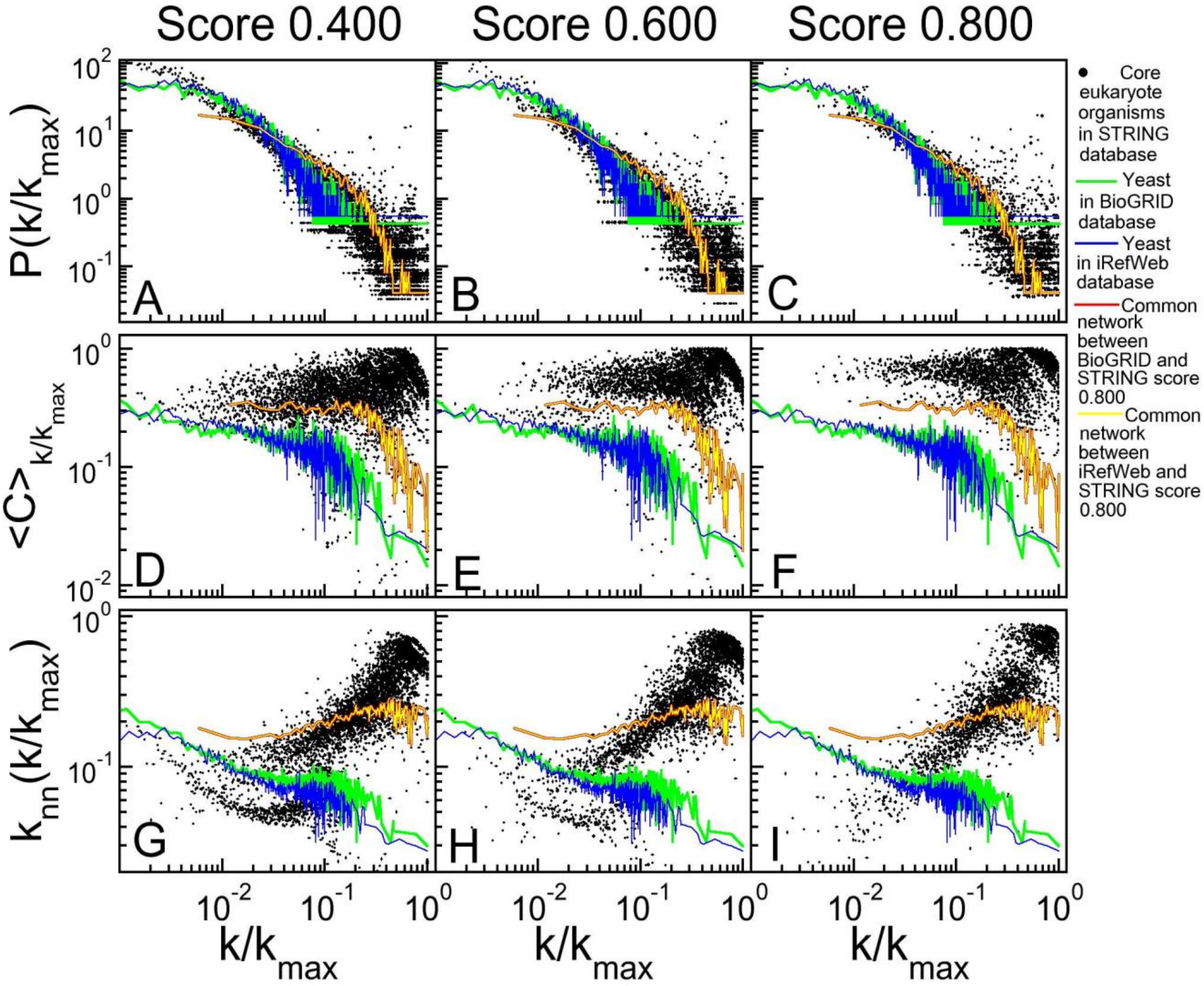

Figure 1. STRING database compared with BioGRIG and iRefWeb yeast networks. Degree distribution, $P\left({k}/{k_{\max}}\right)$, average clustering coefficient, $\langle C\rangle_{k/k_{max}}$, and average degree of nearest neighbors, $k_{nn}\left({k}/{k_{\max}}\right)$, as function of ${k}/{k_{\max}}$, for different networks. Black dots represent networks of 31 core eukaryotes in STRING database, with confidence scores 0.400, 0.600, and 0.800 in the left, middle and right columns, respectively. The green and blue lines are the results for the yeast networks obtained from BioGRID and iRefWeb databases. Red and yellow lines refer to networks produced by removing any link that was not present in STRING score 0.800 network, without adding any other link.

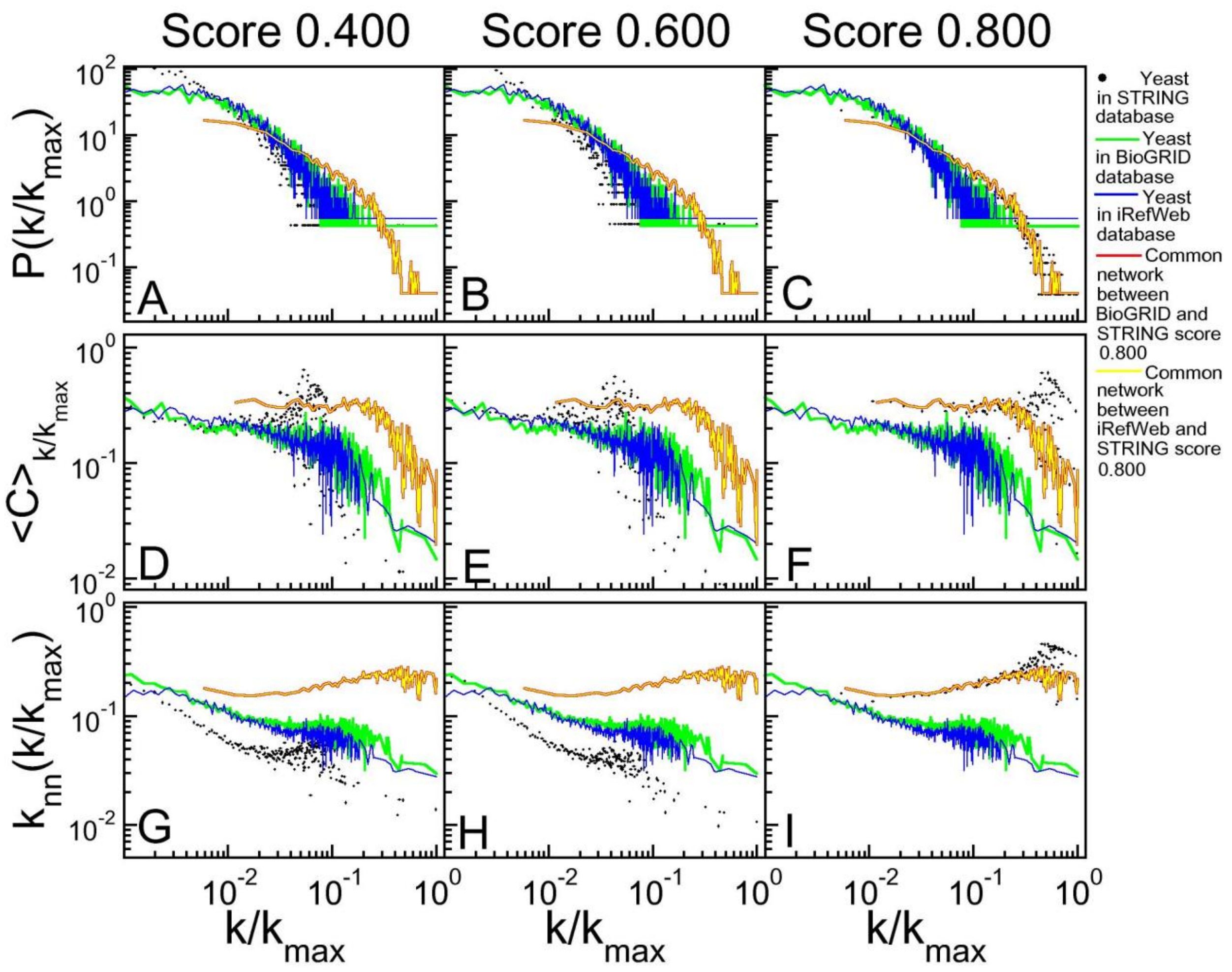

Figure 2. STRING yeast network compared with BioGRIG and iRefWeb yeast networks. Degree distribution, $P\left({}^{k}/_{k_{\max}}\right)$, average clustering coefficient, $\langle C \rangle_{k/_{k_{max}}}$, and average degree of nearest neighbors, $k_{nn}\left({}^{k}/_{k_{\max}}\right)$, as function of ${}^{k}/_{k_{\max}}$, for different networks. Black dots represent yeast network from STRING database, with confidence scores 0.400, 0.600, and 0.800 in the left, middle and right columns, respectively. The green and blue lines are the results for the yeast networks obtained from BioGRID and iRefWeb databases. Red and yellow lines refer to networks produced by removing any link that was not present in STRING score 0.800 network, without adding any other link.

References

1. Stark C, Breitkreutz BJ, Chatr-Aryamontri A, Boucher L, Oughtred R, Livstone MS, Nixon J, Van Auken K, Wang X, Shi X, Reguly T, Rust JM, Winter A, Dolinski K, Tyers M. (2010) The BioGRID Interaction Database: 2011 update. Nucleic Acids Res. **9**(Database issue): D698–D704.

2. Turner B, Razick S, Trinsky AL, Vlasblom J, Crowdy EK, Cho E, Morrison K, Donaldson IM, Wodak SJ (2010) iRefWeb: interactive analysis of consolidated protein interaction data and their supporting evidence. Database (Oxford) 10.1093/database/baq023 [doi].
