# Supplementary material for: Preferential Duplication of Intermodular Hub Genes: An Evolutionary Signature in Eukaryotes Genome Networks
Source: PLoS One. 2013 Feb 26;8(2):e56579. doi: 10.1371/journal.pone.0056579 (PMC3582557; doi:10.1371/journal.pone.0056579)
Supplement: Text S4 — Correlation between network properties and Evolutionary Plasticity Index (EPI). In this text we present and discuss a plot of the average duplication probability for different gene families versus the average Evolutionary Plasticity Index (EPI), in order to further support the model assumption of a duplication probability. (PDF) [file pone.0056579.s005.pdf]

Supplementary material online for

Preferential duplication of intermodular hub genes: an evolutionary signature in eukaryotes genome networks.

Ricardo M. Ferreira*[1],José Luiz Rybarczyk-Filho*[1], Rodrigo J. S. Dalmolin*[3], Mauro A. A. Castro[1,2], José C. F. Moreira[3], Leonardo G. Brunnet[1]& Rita M. C. de Almeida[1,2]

Instituto de Física[1], NationalInstituteof Science and Technology for Complex Systems[2], and Departamento de Bioquímica[3], Universidade Federal do Rio Grande do Sul, Av. Bento Gonçalves, 9500, 91051-970 C.P. 15051, Porto Alegre, Brazil.

***These authors contributed equally to this paper**

Correspondenceto:

Rita M. C. de Almeida
Instituto de Física, Universidade Federal do Rio Grande do Sul,
Av. Bento Gonçalves, 9500, 91051-970 C.P. 15051, Porto Alegre, Brazil.

Correlation between network properties and Evolutionary Plasticity Index (EPI)

To further support the model assumption of a duplication probability given by Eq.5, that is

$$p_i^D = \frac{k_i(1 - C_i)}{\sum_{j=1}^{N} k_j (1 - C_j)},$$

we present a plot for the average duplication probability for different gene families versus the average Evolutionary Plasticity Index (EPI), as defined by Dalmolin*et al.* [1].The 5 metabolic pathways for *Saccharomyces cerevisiae*were generated from KEGG [2]and STRING [3-5] database as described in [1]. RNA Binding protein family is obtained from Mittal *et al.* [6]. There is a clear positive correlation between these two quantities.

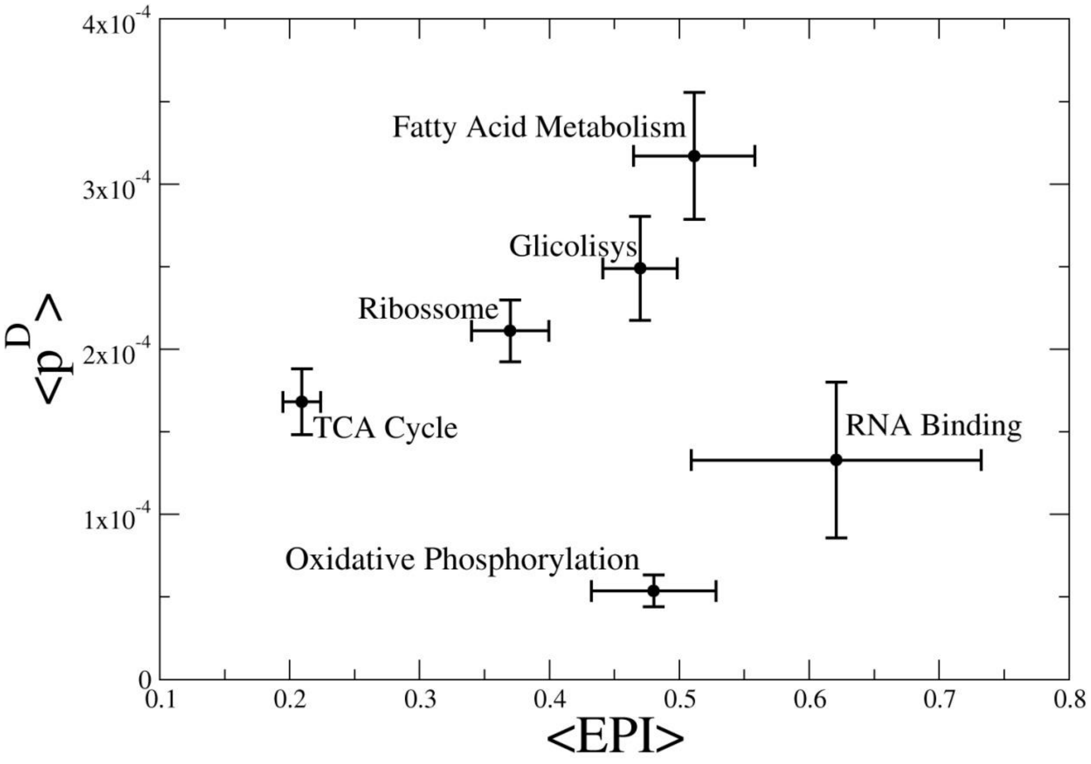

Figure 1 – Averaged duplication probability versus average EPI for different gene families in *Saccharomyces cerevisiae*.

References

1. Dalmolin R, Castro M, RybarczykFilho J, Souza L, de Almeida R, Moreira J (2011) Evolutionary plasticity determination by orthologous groups distribution. Biology Direct 6: 22.

2. Kanehisa, M., Goto, S., Sato, Y., Furumichi, M., and Tanabe, M. (2012) KEGG for integration and interpretation of large-scale molecular datasets. Nucleic Acids Res. 40, D109-D114.

3. Jensen LJ, Kuhn M, Stark M, Chaffron S, Creevey C, Muller J, Doerks T, Julien P, Roth A, Simonovic M, Bork P, von Mering C (2009) STRING 8--a global viewonproteinsandtheirfunctionalinteractions in 630 organisms. NuclAcids Res 37: D412-D416.

4. von Mering C, Jensen LJ, Snel B, Hooper SD, Krupp M, Foglierini M, Jouffre N, Huynen MA, Bork P (2005) STRING: knownandpredictedprotein-proteinassociations, integratedandtransferredacrossorganisms. NuclAcids Res 33: D433-D437.

5. von Mering C, Jensen LJ, Kuhn M, Chaffron S, Doerks T, Kruger B, Snel B, Bork P (2007) STRING 7--recentdevelopments in theintegrationandpredictionofproteininteractions. NuclAcids Res 35: D358-D362.

6. Mittal M, Scherrer T, Gerber AP, Janga SC (2011) Interplay between posttranscriptional and posttranlational interactions of RNA-binding proteins. J. Mol. Biol. 409, 466-479 doi: 10.1016/j.jmb.2011.03.064.
