# Supplementary material for: Preferential Duplication of Intermodular Hub Genes: An Evolutionary Signature in Eukaryotes Genome Networks
Source: PLoS One. 2013 Feb 26;8(2):e56579. doi: 10.1371/journal.pone.0056579 (PMC3582557; doi:10.1371/journal.pone.0056579)
Supplement: Text S5 — Parameters of the models. In this text we discuss the choice of parameters in Barabási-Albert, Duplication-Divergence, and Duplication-Acquisition models. (PDF) [file pone.0056579.s006.pdf]

## Supplementary material online for

# Preferential duplication of intermodular hub genes: an evolutionary signature in eukaryotes genome networks.

Ricardo M. Ferreira*[1],José Luiz Rybarczyk-Filho*[1], Rodrigo J. S. Dalmolin*[3], Mauro A. A. Castro[1,2], José C. F. Moreira[3], Leonardo G. Brunnet[1]& Rita M. C. de Almeida[1,2]

Instituto de Física[1], NationalInstituteof Science and Technology for Complex Systems[2], and Departamento de Bioquímica[3], Universidade Federal do Rio Grande do Sul, Av. Bento Gonçalves, 9500, 91051-970 C.P. 15051, Porto Alegre, Brazil.

***These authors contributed equally to this paper**

**Correspondenceto:**

Rita M. C. de Almeida
Instituto de Física, Universidade Federal do Rio Grande do Sul,
Av. Bento Gonçalves, 9500, 91051-970 C.P. 15051, Porto Alegre, Brazil.

**Parameters of the models**

In figs. 1-4 we change the model parameters and see how they affect the results. Duplication-Acquisition model has two parameters to explore. We first varied *q*, that rules the fraction of duplicated nodes,and presented the results in Fig. 1, where we can see that for decreasing the number of duplicated nodes (smaller *q*) the network approaches the ones generated using Barabási-Albert algorithm (Fig. 3). Thiswas expected, since the other mechanism for adding nodes is similar to B-A model. In Fig. 2 we present the results for varying *r*, the parameter responsible for controlling mutation. For small *r*, the distributions resemble the ones obtained through Duplication-Divergence (Fig. 4) model namely: $k_{\max}$ , clustering, and nearest neighbor degree decrease.

We changed parameter *m* forBarabási-Albert model [1] and can see in Fig. 3 that the results remain almost the same. In Fig. 4, we can see that as we increase the parameter *p* in Duplication-Divergence model [2,3], which controls the divergence, the maximum degree decreases, its degree distribution approaches a power law for large values of $k/k_{\max}$, and clustering and mean degree of nearest neighbors do not have significant changes. Finally, the number of links significantlydiffers from the six well studied organisms, presented in orange in figure (c).

.

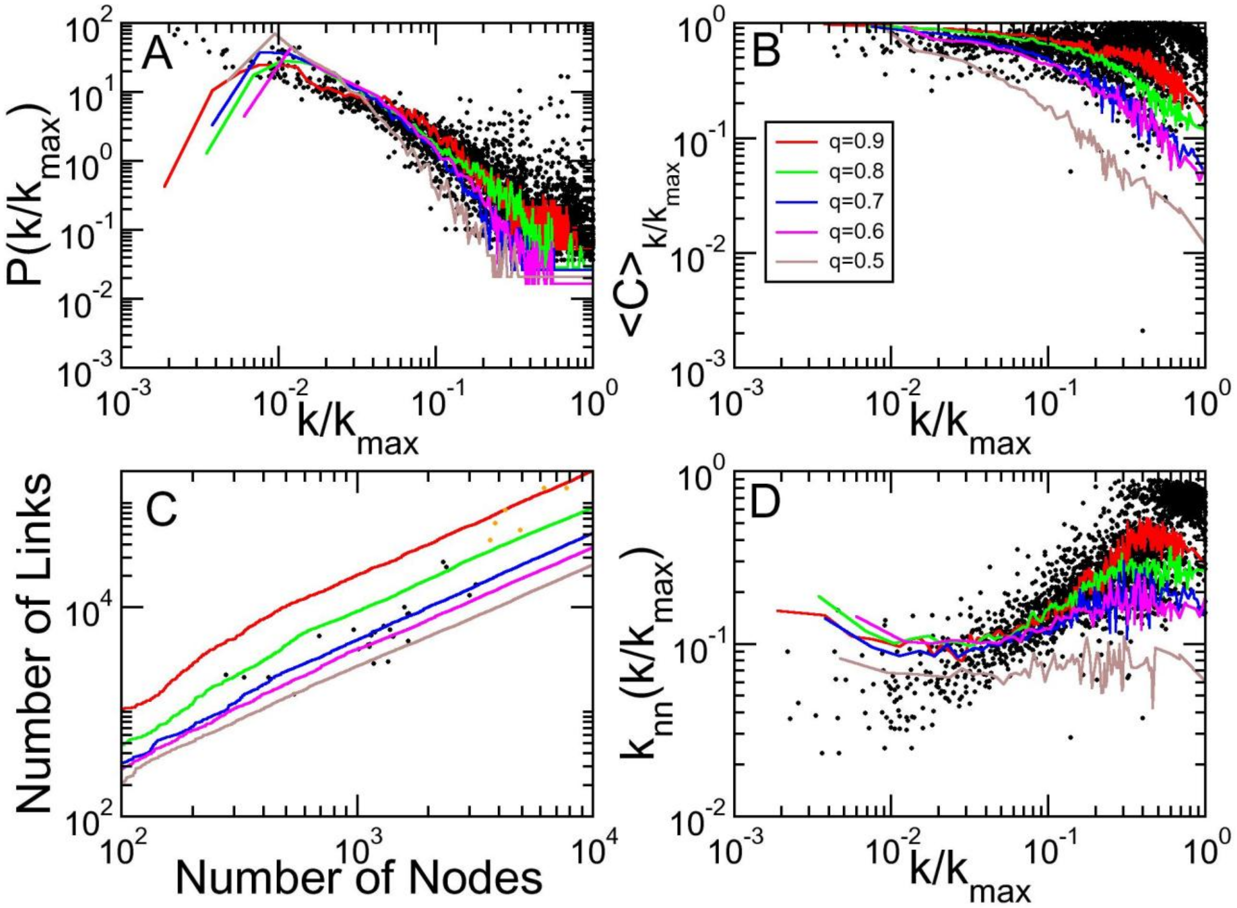


**Figure 1.Five networks obtained using the Duplication-Acquisition model**. Different values of parameter $q$, which determines the fraction of nodes acquired by duplication, maintaining constant $r$, the mutation probability. The black dots represent  the networks for all 31 core eukaryote organisms, with confidence score 0.800. We can see that, as the number of acquired nodes increases, the network approaches a Barabási-Albert one, as we can see in Figures (**a**), (**b**), and (**d**). Namely, the network loses the high probability of finding high degree nodes in the degree distribution (Figure (**a**)), the clustering coefficient decreases (Figure(**b**)), and the network loses its assortativity (Figure (**d**)).  Figure (**c**) shows that the number of links also decreases, falling below the value presented by organisms.

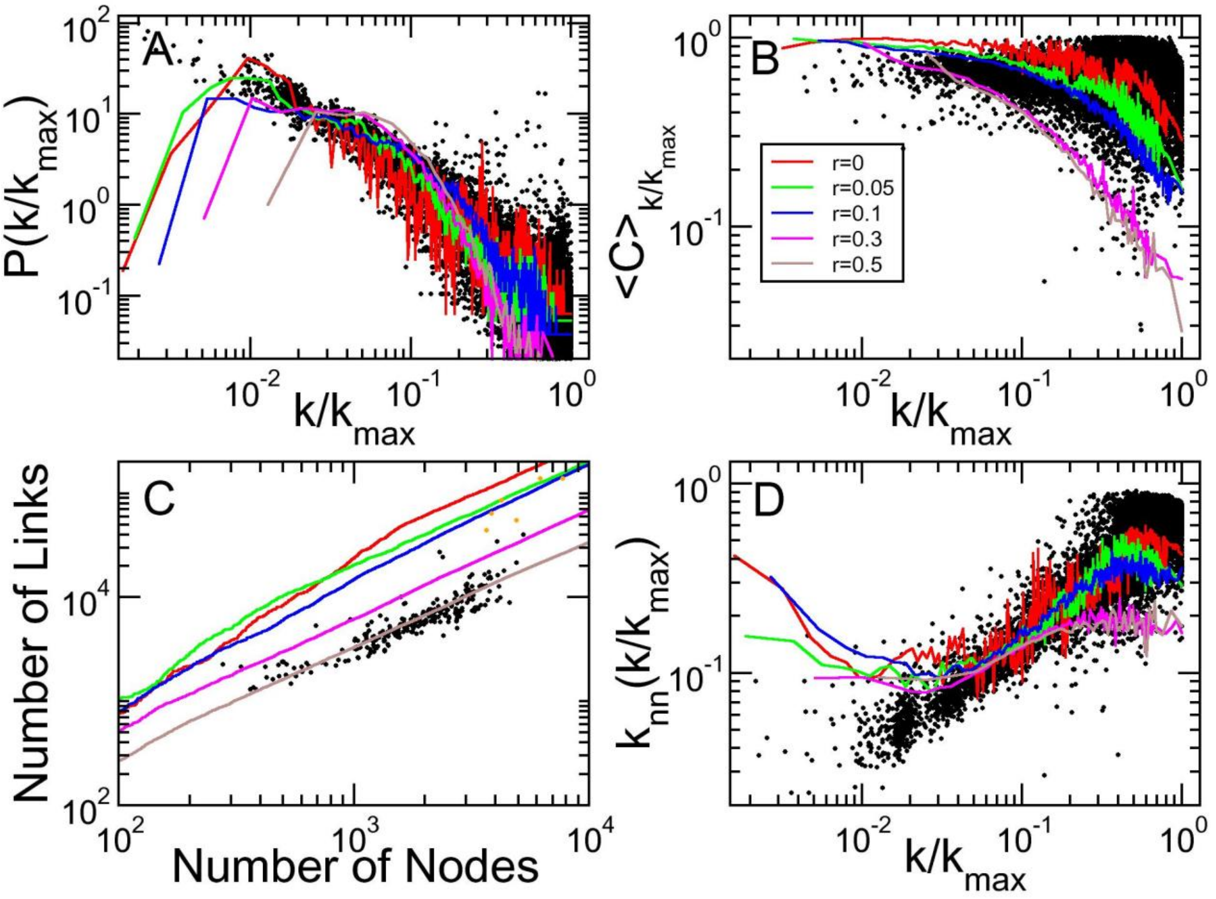


**Figure 2. Seven networks obtained using the Duplication-Acquisition model.**Different values of parameter *r*, which determines the mutation probability, maintaining constant *q*, the fraction of nodes acquired by duplication. Black dots represent networks for all 31 core eukaryote organisms, with confidence score 0.800. We can see that, as mutation probability increases, the network approaches the ones obtained using the Duplication-Divergence model. In Figure (a) the degree distribution approaches a power-law, and in (b) the clustering coefficient decreases. Figure (c) shows that the number of links decreases, and in Figure (d) present the mean nearest degree distribution for the networks.

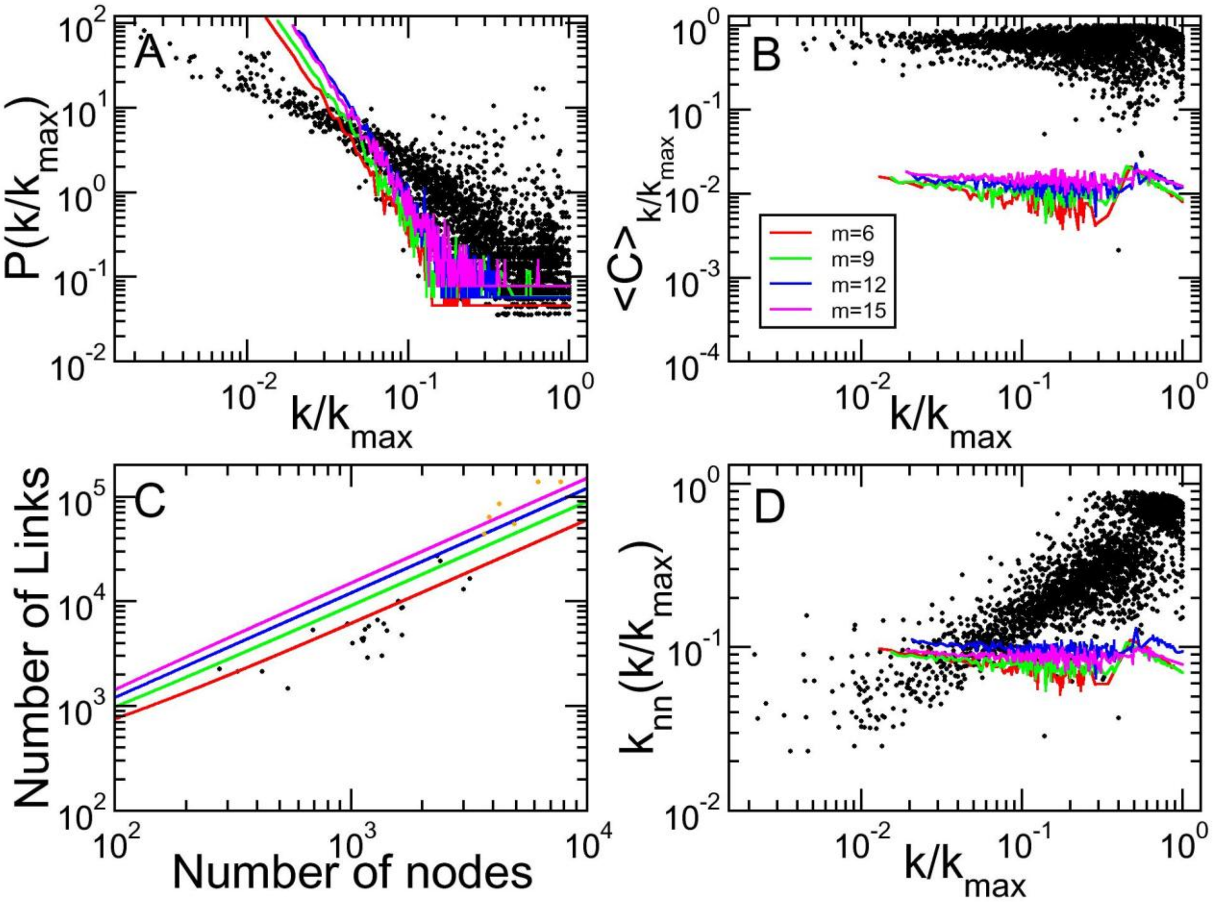


**Figure 3. Four networks obtained using Barabási-Albert model.** Different values of parameter *m*, which determines the number of links of each new node. Black dots represent networks for all 31 core eukaryote organisms, with confidence score 0.800. In Figure (a) we can see that the degree distribution follows a power-law and does not correctly represent the degree distribution of the organisms networks. Figure (b) shows that clustering coefficient of the simulated networks is lower than the experimental data. Figure (c) presents the evolution of number of links with number of nodes. Figure (d) shows that the average degree of the neighbors of a node is independent of the node degree for networks built using Barabási-Albert model, what deviates from the behavior presented by the organisms networks that are highly assortative.

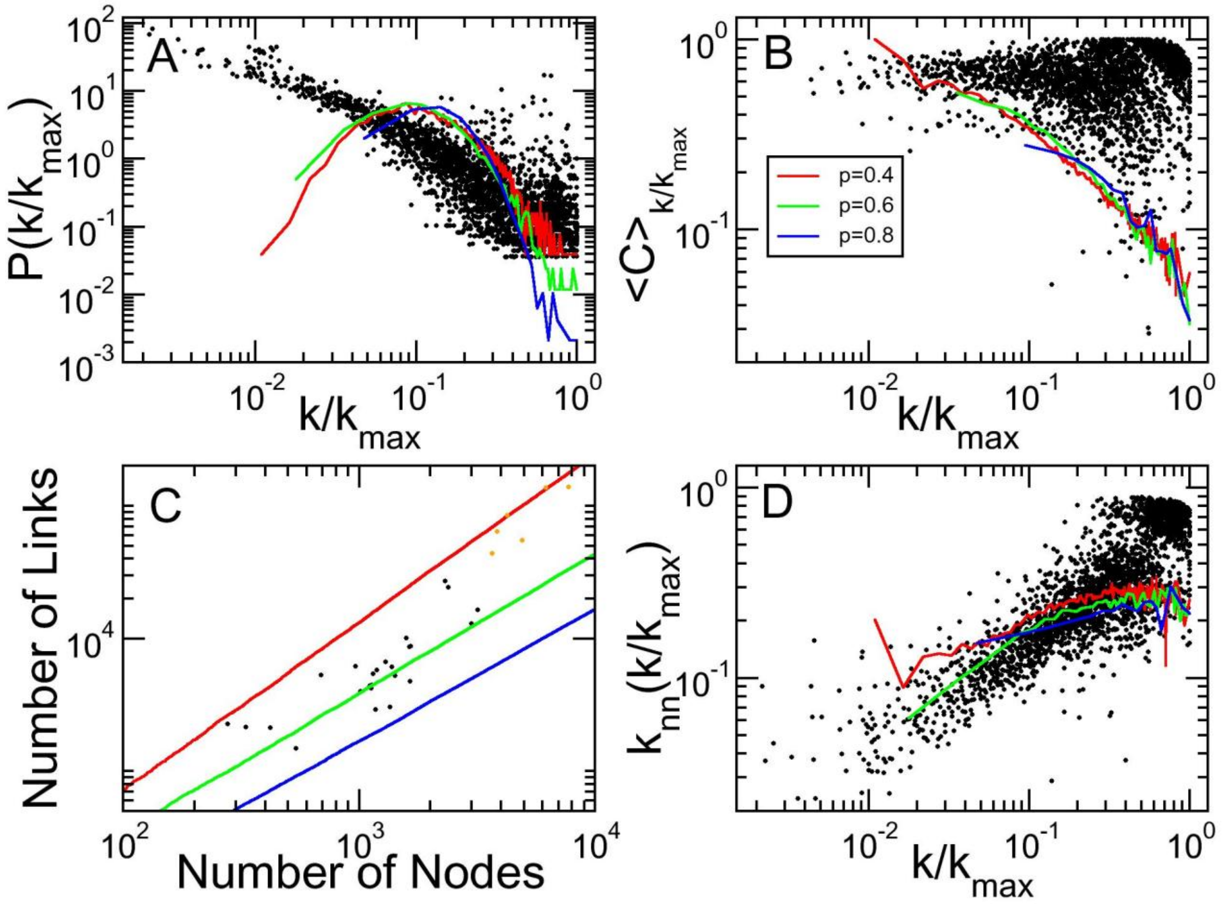


**Figure 4.Three networks obtained using Duplication-Divergence model.** Different values of parameter *p*, which determines the mutation probability. The black dots represent networks for all 31 core eukaryote organisms, with confidence score 0.800. In Figure (**a**) we can see that for higher values of *p* the network approaches a power-law, but as we can see in Figure (**c**), the number of links fall below those found for the organisms. Figure (**b**) shows that the clustering decreases with degree. In Figure (**d**) we have the average degree of nearest neighbors, which increases with degree, showing that the Duplication Divergence model builds networks with the same assortativeness of the organisms.

## References

1. Barabasi AL, Albert R (1999) Emergenceofscaling in random networks. Science 286: 509-512. 7898 [pii].

2. Vázquez A, Flammini A, Maritan A, Vespignani A (2003) ModelingofProteinInteraction Networks. Complexus 1: 38-44.

3. Vázquez A (2003) Growing network with local rules: preferentialattachment, clusteringhierarchy, anddegreecorrelations. PhysRev E: StatNonlin Soft MatterPhys 67: 056104.
