# Supplementary material for: Preferential Duplication of Intermodular Hub Genes: An Evolutionary Signature in Eukaryotes Genome Networks
Source: PLoS One. 2013 Feb 26;8(2):e56579. doi: 10.1371/journal.pone.0056579 (PMC3582557; doi:10.1371/journal.pone.0056579)
Supplement: Text S6 — Ordering Algorithm. In this text we further discuss the ordering algorithm, and present some properties of networks that can be evinced with this algorithm. (PDF) [file pone.0056579.s007.pdf]

Supplementary material online for

Preferential duplication of intermodular hub genes: an evolutionary signature in eukaryotes genome networks.

Ricardo M. Ferreira*[1],José Luiz Rybarczyk-Filho*[1], Rodrigo J. S. Dalmolin*[3], Mauro A. A. Castro[1,2], José C. F. Moreira[3], Leonardo G. Brunnet[1]& Rita M. C. de Almeida[1,2]

Instituto de Física[1], NationalInstituteof Science and Technology for Complex Systems[2], and Departamento de Bioquímica[3], Universidade Federal do Rio Grande do Sul, Av. Bento Gonçalves, 9500, 91051-970 C.P. 15051, Porto Alegre, Brazil.

***These authors contributed equally to this paper**

Correspondenceto:

Rita M. C. de Almeida
Instituto de Física, Universidade Federal do Rio Grande do Sul,
Av. Bento Gonçalves, 9500, 91051-970 C.P. 15051, Porto Alegre, Brazil.

Ordering Algorithm.

Figures S1-S3 present the association matrices for different networks, where some topological properties are evinced by the ordering algorithm [1]. In Fig. S1 we orderedErdös-Rényiwith$\alpha = 1$,to show that the black dots, which represent links between proteins, are spread all over the matrix, without presenting any module. Figure S2 shows the central part of the orderings presented in Fig. 4with $\alpha = 8$, in the main text. These regions, which in the complete ordering of the organisms networks appear to be uniformly dense, in fact present a very modular structure. Also, we can see that this modular structure is much better reproduced in D-A Model than in B-A and D-D Models. Finally, in Figure S3 we present the orderingswith$\alpha = 1$, where we can clearly see the modular structure in the organisms networks. D-D Model does not present this structure with small modules, and B-A Model just presents a central cross, which stems from the central hub. D-A Model presents the modular structure along the diagonal, but also a faint cross from the nodes acquired *de novo*.

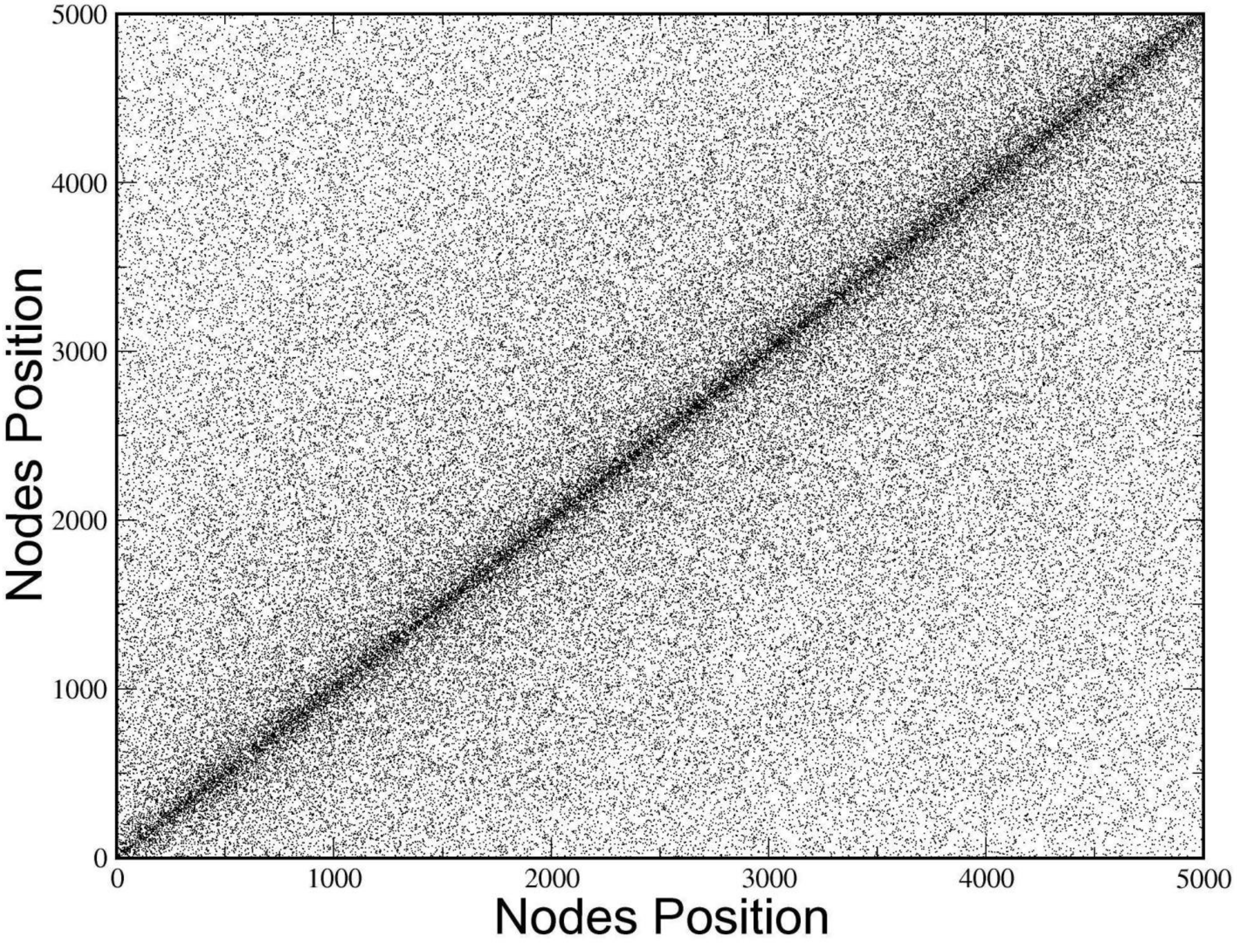

Figure S1. Association matrix for a Erdös-Rényinetwork . 5000 nodes and 63025 links, ordered using α=1.

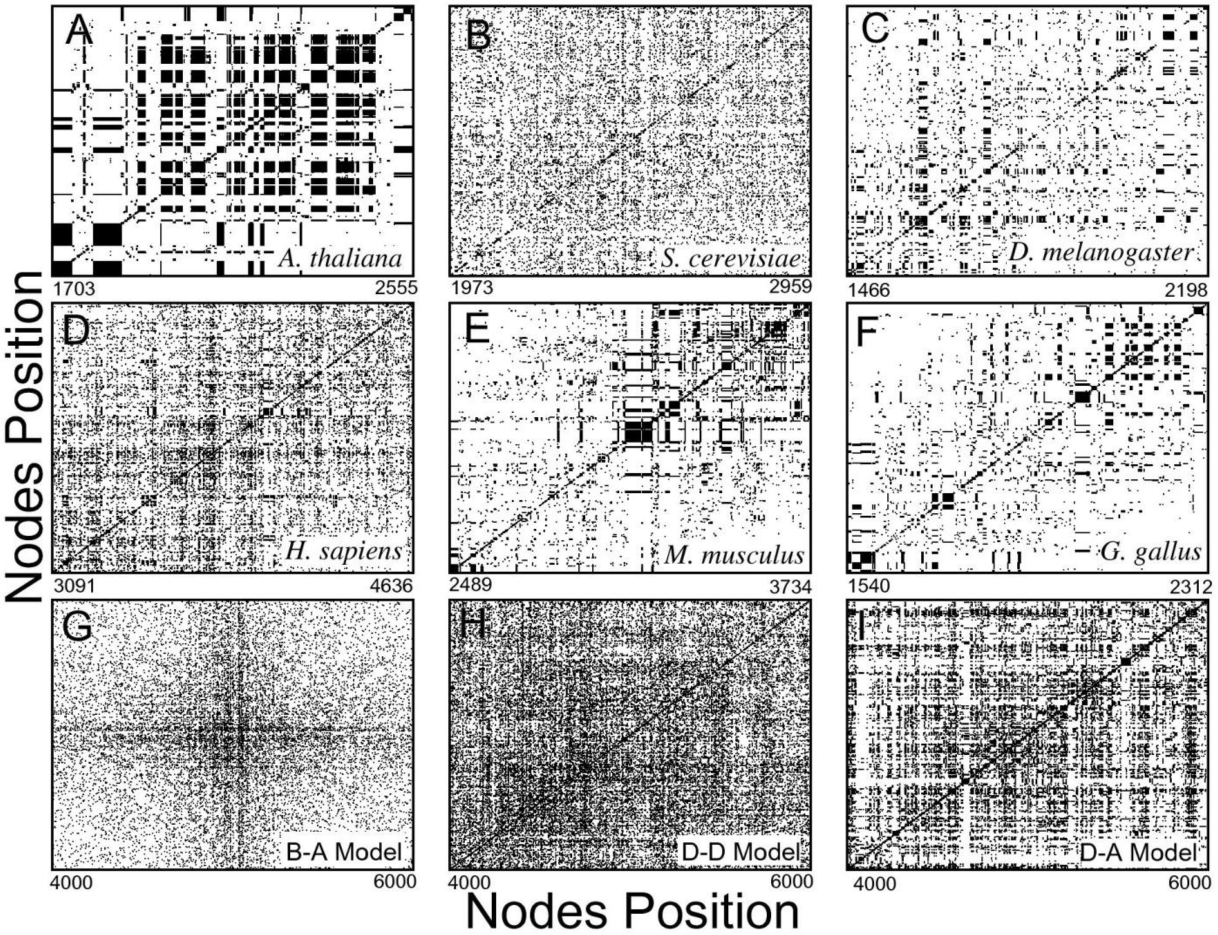

Figure S2. Zoom at the central part of association matrices in Fig.3. From 0.4N to 0.6 N, for **(a)** *Arabidopsis thaliana*,**(b)** *Saccharomyces cerevisiae***,** **(c)** *Drosophila melanogaster*, **(d)** *Homo sapiens*, **(e)** *Musmusculus*, **(f)** *Gallus gallus*,**(g)**Barabási-Albert model, **(h)** duplication-divergence model and **(i)** duplication-acquisition model, ordered using α=8.

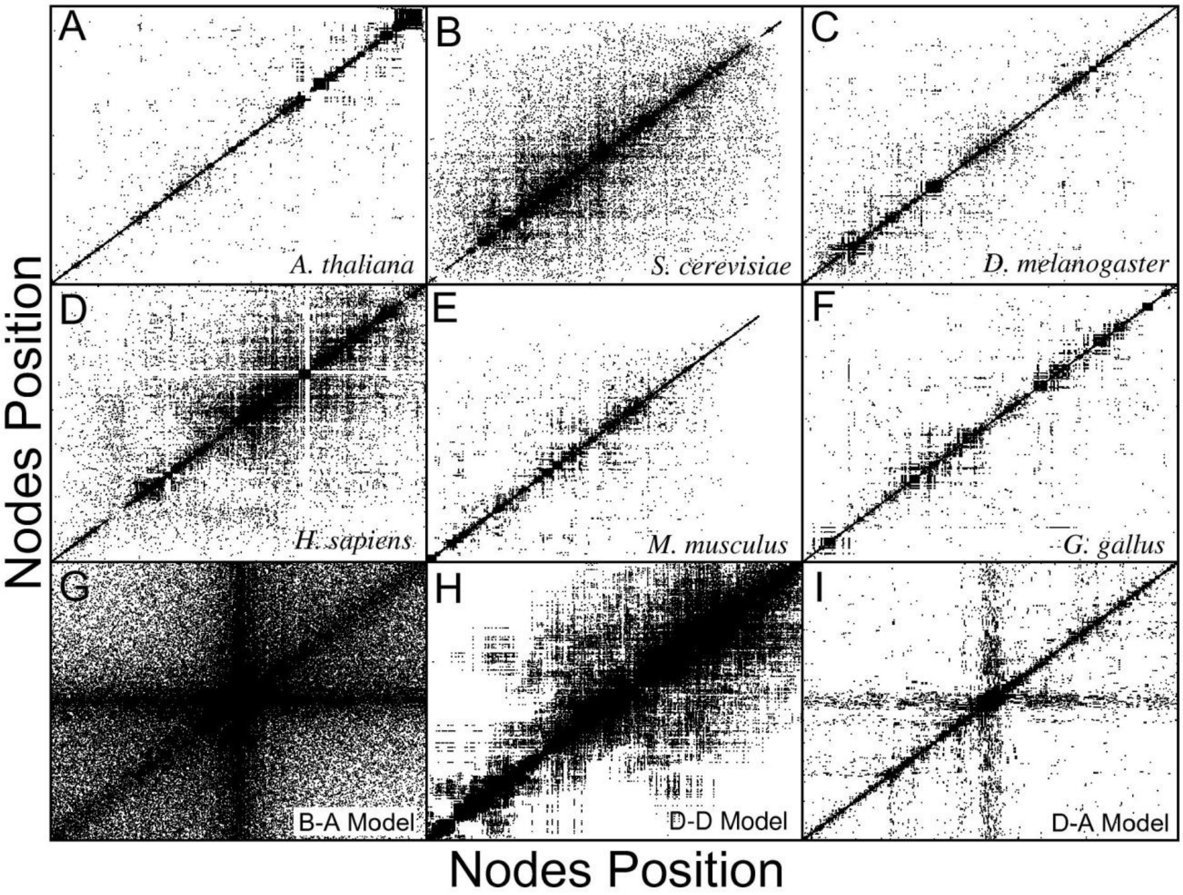

Figure S3. Association matrices.(a) *Arabidopsis thaliana*,**(b)** *Saccharomyces cerevisiae***, (c)** *Drosophila melanogaster,* **(d)** *Homo sapiens,* **(e)** *Musmusculus,* **(f)** *Gallus gallus*,**(g)**Barabási-Albert model, **(h)** duplication-divergence model and **(i)** duplication-acquisition model, ordered using α=1.

References

1. Rybarczyk-Filho JL, Castro MA, Dalmolin RJ, Moreira JC, Brunnet LG, de Almeida RMC (2010) Towards a genome-widetranscriptogram: the*Saccharomycescerevisiae* case. NucleicAcids Res .gkq1269 [pii];10.1093/nar/gkq1269 [doi].
